# An innervated and vascularized HNSCC-on-a-chip model built on defined and tunable engineered extracellular matrices

**DOI:** 10.1073/pnas.2602541123

**Published:** 2026-10-27

**Authors:** Alessia Maria Pignataro, Cornelia Christina Schwarz, Emilia Wiechec, Alessandro Cordiale, Shyama Sasikumar, Alexander Jenssen, Taka Ariyaberg, Lalit Pramod Khare, Ehsanul Hoque Apu, Karin Roberg, Sajjad Naeimipour, Gabriela Basile Carballo, Marcin Szczot, Daniel Aili, Marco Rasponi, Pierfrancesco Pagella

**Affiliations:** ^a^Laboratory of Molecular Materials, Division of Biophysics and Bioengineering, Department of Physics, Chemistry and Biology, Campus Valla, Linköping 58330, Sweden; ^b^ MiMic Lab, Department of Electronics, Information and Bioengineering, Politecnico di Milano, Milano 20133, Italy; ^c^ Division of Cell and Neurobiology, Department of Clinical and Biomedical Sciences, Linköping University, Linköping 58183, Sweden; ^d^ Region Östergötland Anesthetics, Operations and Specialty Surgery Center, Department of Otorhinolaryngology, Linköping 582 25, Sweden; ^e^ Bitonte College of Dentistry, Department of Biomedical Sciences, Northeast Ohio Medical University, Rootstown, OH 44272; ^f^ Department of Biomedical and Clinical Sciences, Center for Social and Affective Neuroscience, Linköping University, Linköping 58183, Sweden

**Keywords:** oral cancer, organ-on-a-chip, extracellular matrix, innervation, vasculature

## Abstract

Understanding the tumor microenvironment requires experimental platforms that faithfully recapitulate its key components. Here, we present an innervated and vascularized head and neck squamous cell carcinoma (HNSCC)-on-a-chip platform built with fully defined and tunable engineered extracellular matrices (eECMs). In a stepwise increase of complexity, we first cocultured patient-derived HNSCC cells, cancer-associated fibroblasts, and endothelial cells within tailored eECMs, revealing extracellular matrix-dependent differences in transcriptional state, multicellular self-organization, and chemotherapeutic sensitivity. We then integrated these three-dimensional constructs into a cancer–vasculature interface, which enabled analysis of eECM-dependent directional collective migration and invasion. Finally, we incorporated trigeminal innervation through injectable 3D human bioengineered trigeminal ganglia, establishing a chip-based innervation–tumor–vasculature tri-interface. Together, this all-human platform integrates fully defined ECM, vasculature, and innervation in a single modular system to investigate tumor organization, invasion, and therapeutic responses within a controlled human microenvironment.

## Significance

Understanding solid tumors requires models that capture the combined influence of extracellular matrix, vasculature, and innervation on tumor behavior. To this end, we present a fully human organ-on-a-chip model of head and neck squamous cell carcinoma integrating tumor tissue, an endothelialized vascular compartment, and stem cell–derived trigeminal ganglia within defined and tunable engineered extracellular matrices. Transcriptomic and imaging analyses demonstrate that extracellular matrix composition profoundly shapes tumor architecture and transcriptional state, while the vascular and neural components enable investigation of tumor–vascular interactions, invasion, therapeutic responses, and innervation-associated changes in tumor organization. Together, these findings establish a versatile human platform for investigating tumor–matrix–vascular–neural interactions and evaluating therapeutic responses in complex engineered microenvironments.

The need to study human cancers in their complexity and patient-specificity is driving the generation of all-human 3D preclinical models. While in vivo models provide complex emulation platforms for human cancers, they raise significant ethical concerns, and they fall short in replicating human- and patient-specific features (1). Among in vitro emulation systems, organ-on-a-chip devices are gaining a prominent role in the generation of complex human disease models. These devices are designed to control cell microenvironments and maintain tissue-specific functions, aiming to mimic human physiology (2). Early organ-on-a-chip systems connected parallel microchannels separated by a porous membrane to mimic tissue–tissue interfaces. Since then, diverse microfluidic architectures have emerged that achieve similar compartmentalization without membranes, e.g., designs that use micropillars to define central culture chambers or incorporate biological materials to reproduce natural barriers (3). This arrangement recreates tissue–tissue interfaces and closely emulates the minimal functional units found in tissues and organs. The devices allow for real-time, high-resolution imaging of cultured cells and tissues. Moreover, the vascular channels of different organ-specific chips can be linked to study multiorgan physiological coupling (2–5). In the context of cancer, organ-on-a-chip platforms allow real-time observation of tumor–immune interactions, drug responses, and cellular behavior under conditions that closely resemble in vivo settings. This is particularly valuable for studying cancer cell behavior and testing therapeutic interventions in a controlled yet biologically meaningful environment (2).

Head and neck squamous cell carcinoma (HNSCC) is the sixth most common cancer worldwide with nearly a million new cases registered in 2020 (6). Despite the progress in treatment strategies, advanced HNSCC remains associated with a harsh prognosis (5-y overall survival, <50 %), with a high risk of distant metastasis and local recurrence (6). Current therapies for HNSCC include surgery, radiation therapy, chemotherapy, targeted therapy (notably cetuximab, an EGFR inhibitor), and immunotherapy with immune checkpoint inhibitors such as pembrolizumab and nivolumab (7). Although these treatments have improved outcomes for some patients, their overall efficacy remains limited, as indicated by the high mortality rate (8). Significant barriers persist in reaching a comprehensive understanding of the biology of HNSCC and treatment responses. These limitations primarily arise from the lack of appropriate experimental models that can capture the complexity of HNSCC and its microenvironment (9). HNSCC is a complex tissue ecosystem where many different cell types, factors, and extracellular matrix interact (10). Key players include cancer-associated fibroblasts (CAFs), vasculature, innervation, and the extracellular matrix (ECM). CAFs stimulate tumor growth, epithelial-to-mesenchymal transition (EMT), invasive potential, and metastasis, and they also modulate the drug sensitivity of cancer cells (11–13). Vascularization is fundamental in regulating HNSCC progression: while blood supply provides trophic support to the tumor and a route for metastasis, hypoxia within HNSCC regions distant from vascularization appears to facilitate progression by stimulating radio-resistance (14, 15). In recent years, several studies have unraveled the fundamental role of innervation in cancer progression, and in particular, of trigeminal innervation in HNSCC pathogenesis. HNSCC harbors significantly higher neuronal and electrical activity than the corresponding healthy tissue, and innervation actively promotes HNSCC progression and invasiveness (16, 17). Furthermore, trigeminal innervation conveys pain, a common and debilitating feature of HNSCC that often appears as one of the first signs of cancer progression (18). The extracellular matrix (ECM) itself plays a crucial, yet frequently overlooked role in the progression and treatment resistance of HNSCC. Beyond providing structural support, the ECM shapes tumor behavior by influencing cell signaling, migration, and immune evasion (19). In HNSCC, aberrant ECM remodeling contributes to a dense, fibrotic tumor microenvironment that can act as a physical and biochemical barrier to therapeutic agents. This is particularly relevant for drug penetration, as the ECM can hinder the uniform distribution of chemotherapeutics and targeted treatments within the tumor mass (20). Furthermore, the ECM poses a significant obstacle to the infiltration and efficacy of immune cells and CAR-T cells, which have shown promise in hematologic malignancies but face limited success in solid tumors (20, 21).

Recent work has established a small but growing set of HNSCC-on-a-chip and oral-cancer-on-a-chip systems (9, 22). Early studies demonstrated that patient-derived HNSCC biopsies could be maintained ex vivo on-a-chip for days while exposed to chemotherapeutic agents such as cisplatin and 5-FU, preserving viability and enabling dynamic monitoring of apoptosis and proliferation (23). Subsequent platforms extended this approach to radiation testing, showing that perfused tumor slices could reproducibly model dose-dependent cytotoxicity and radiosensitivity in individualized assays (24, 25). Other devices refined perfusion design to sustain small tumor fragments for 48 h while preserving their histoarchitecture (26) or enabled HNSCC slices to undergo radiotherapy and cisplatin treatment (27). More recently, immunocompetent chips have emerged, such as a humanized 3D microfluidic coculture combining HNSCC cells, immune cells, and patient serum (28). In other works, perfused HNSCC biopsy chips were used to assess transcriptomic and secretome responses after irradiation (29), and more recently, oral-cancer on-chip models were proposed to study HNSCC invasion into blood vessels (30) and metastasis into the bone (22).

Building on these advances, we propose four decisive innovations to address fundamental challenges that remain unresolved in the emulation of the TME. First, we introduce an organ-on-a-chip platform that enables the simultaneous recapitulation of three essential TME components: a three-dimensional patient-derived CAF-HNSCC tumor mass, human trigeminal innervation, and a 3D HNSCC–vasculature interface. Second, we use fully defined engineered extracellular matrices (eECMs) with tunable stiffness, composition, and degradability. This represents a critical advance for TME modeling, as existing HNSCC systems, both on-chip and off-chip, either rely on complex yet poorly defined matrices such as Matrigel® or Myogel, or on chemically defined but overly simplified eECMs, including collagen or fibrin. Third, we establish a 3D coculture system integrating patient-derived HNSCC cells, CAFs, and endothelial cells, thereby partially recapitulating tumor heterogeneity while leveraging the extensive global biobanks of patient-derived HNSCC and CAF lines. Finally, we develop 3D and injectable bioengineered human trigeminal ganglia derived from human embryonic stem cells (hESCs) to model oral cancer–specific innervation.

In a stepwise increase of complexity, we first showed that different eECMs induce radically different transcriptional programs in cocultured HNSCC cells, CAFs, and endothelial cells. We then integrated these 3D HNSCC models into a first organ-on-a-chip device that models the cancer–vasculature interface. We observed ECM-driven differential self-organization of carcinoma cells, collective migration, and vascular invasion. ECM and model complexity strongly influenced the response of 3D HNSCCs to cisplatin, a commonly used chemotherapeutic. We then implemented hESC-derived bioengineered trigeminal ganglia in the complete organ-on-a-chip device that models innervation/cancer and cancer/vascular interfaces. We observed rapid and abundant innervation of HNSCC/CAFs, and innervation-dependent changes in tumor size and collective migration. Taken together, our study proposes an innovative innervated and vascularized 3D model of HNSCC, developed in completely defined extracellular matrices with tunable stiffness, composition, and degradability, which allows fine control of the tumor microenvironment and permits the study of HNSCC behavior at the tumor–vasculature and tumor–innervation interfaces. The platform can be further easily adapted to the investigation of biological questions in which controlled and defined 3D eECM, innervation, and vasculature are needed in an in vitro or ex vivo setting.

## Results

### Fabrication of an Organ-on-a-Chip Device for the Emulation of the Interface Between HNSCC, Vasculature, and Trigeminal Innervation.

**Fig. 1.**
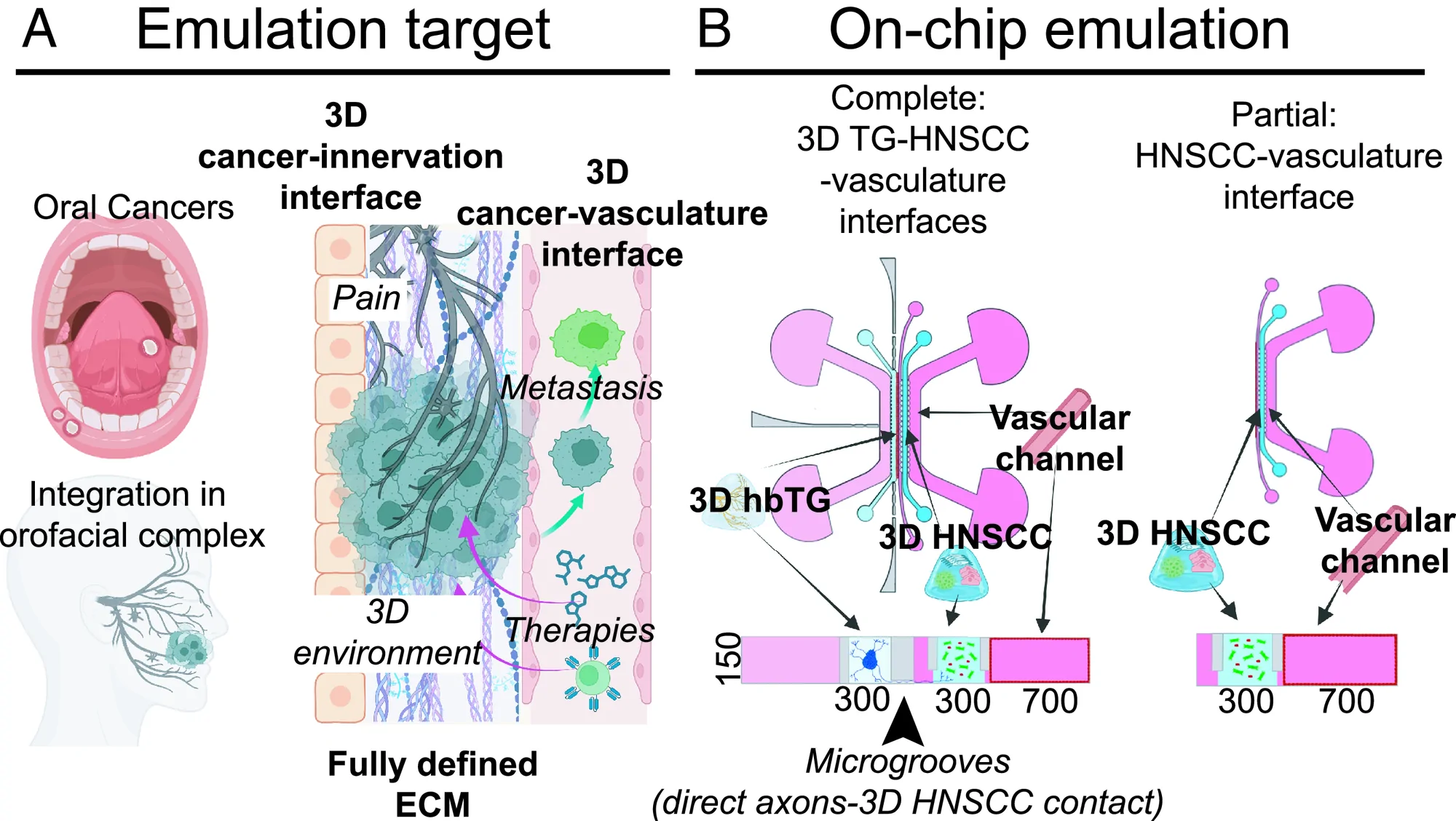
Organ-on-a-chip device for the emulation of the interface between HNSCC, vasculature, and trigeminal innervation. (*A*) Schematic representation of the emulation target of the HNSCC-on-a-chip device. (*B*) Basic design of the Complete HNSCC-on-a-chip device (*Left*) and Reduced HNSCC-on-a-chip device (*Right*).

We generated an organ-on-a-chip device that allows the emulation of the 3D interface between HNSCC tumor mass, vasculature, and trigeminal innervation (Fig. 1*A*). The device is composed of four adjacent, sequential chambers (Fig. 1*B*): in the middle, two chambers for the 3D culture of HNSCC and human bioengineered trigeminal ganglia (hbTG), on the right a vascular chamber, on the left a channel to supply medium to the hbTG (Fig. 1*B*). The 3D HNSCC chamber is separated from the adjacent vascular chamber by micropillars (*SI Appendix*, Fig. S1*A*). These micropillars allow containment of the hydrogel-cells mixture (3D HNSCC) during the crosslinking phase. The pillars are discontinuous to permit direct contact and formation of a continuous endothelial barrier between the 3D HNSCC and the vascular chamber upon seeding of endothelial cells in the latter. Culture medium for the 3D HNSCC is supplied via the vascular chamber. The 3D HNSCC chamber is connected to the 3D trigeminal chamber via microgrooves (5 × 10 × 150 μm, 50 μm spacing; *SI Appendix*, Fig. S1*A*) that allow separation of media between the two compartments and the passage of axons, but not of whole cells. This setup aims to reproduce the anatomical separation between the soma of trigeminal neurons, located in the trigeminal ganglion, and the innervation of HNSCC that occurs in the oral cavity. The 3D trigeminal ganglia are supplied with culture medium via a dedicated medium channel, located on the extreme left side of the chip (Fig. 1*B*). We further produced a simplified version of the device that includes only the 3D HNSCC and the vascular chamber, for experiments focused on the tumor–vasculature interface without inclusion of trigeminal innervation (Fig. 1*B*). To characterize molecular transport across the different compartments of the microfluidic devices, we performed diffusion assays using fluorescent tracers of different molecular weights (Rhodamine B, 500 Da; Dextran, 2,000 kDa; (*SI Appendix*, Fig. S1*B*). In both the partial and complete device configurations, Rhodamine rapidly diffused across the compartment interfaces, whereas Dextran remained largely confined to the medium channels throughout the experiment. These results demonstrate selective molecular transport across the engineered interfaces while maintaining effective compartmentalization of large molecules.

### The Composition of the Engineered Extracellular Matrix (eECM) Induces Distinct Transcriptional and Phenotypic States in Cocultured HNSCCs, CAFs, and Endothelial Cells.

**Fig. 2.**
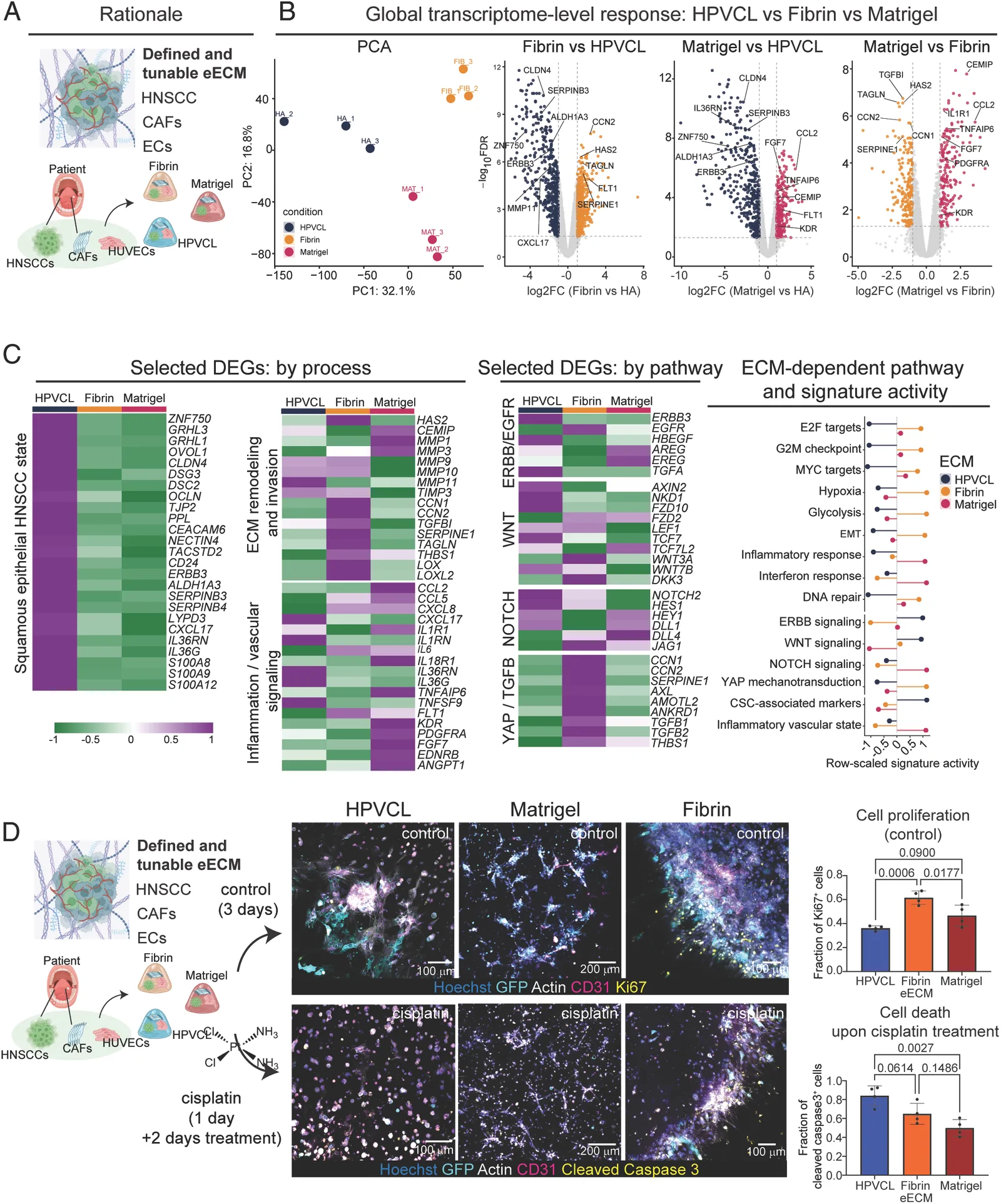
Emulating the HNSCC/ECM interface: coculture of HNSCC cells, cancer-associated fibroblasts (CAFs), and endothelial cells (HUVEC) in different extracellular matrices (ECM). (*A*) Experimental rationale. (*B*) Global transcriptional response to extracellular matrix composition. N = 3 per condition; each replicate consists of a pool of four technical replicates. Principal component analysis (PCA) and differential gene expression analysis of HCHs cultured in the three extracellular matrices. Threshold for volcano plots: log2 fold change > |1|; adjusted *P* < 0.05. (*C*) Heatmaps summarizing extracellular matrix-dependent transcriptional programs. Pathway and signature activity scores derived from Hallmark gene sets and custom HNSCC-related signatures summarize the dominant biological programs associated with each extracellular matrix. (*D*) Representative immunofluorescence images and quantification of HCH proliferative activity (Ki67), and of cisplatin-induced apoptosis (cleaved caspase-3) in HCHs cultured in HPVCL, fibrin, or Matrigel. Data are shown as individual biological replicates (N = 4 per condition) with mean ± SD. Statistical significance was assessed using one-way ANOVA followed by Tukey’s multiple-comparison testing.

We first assessed the effect of different engineered extracellular matrices (eECMs) on the behavior of HNSCC cells (HNSCCs), cancer-associated fibroblasts (CAFs), and endothelial cells (HUVECs), collectively referred to as HCHs, in isolated 3D dome cultures (Fig. 2*A*). For this purpose, we generated a fully defined, complex eECM with the above-mentioned properties by modifying hyaluronan with bicyclo[6.1.0]non-4-yn-9-ylmethyloxycarbonyl]-1,8-diamino-3,6-dioxaoctane (HA-BCN), allowing for cross-linking using azide-terminated peptides and linear and multiarm poly(ethylene glycol) (PEG-Azn) by strain-promoted alkyne–azide cycloaddition (SPAAC). We selected HA-BCN and 8-arm PEG-Azide (PEG-Az8) concentrations and ratios that provide a relatively stiff matrix suitable for 3D culture, based on previous characterization of this hydrogel system (31, 32). The modular HA-BCN/PEG-Azide chemistry enables hydrogel stiffness to be tuned by varying PEG functionalization and/or component concentrations (31, 32). We further included the protease-degradable crosslinker VPM-Az2 to enable cell-mediated matrix remodeling, and collagen I and recombinant human laminin as natural components of the tumor ECM (HA/PEG/VPM/ColI/Lam: HPVCL; Fig. 2*A*). Cross-linking of the hydrogel commences immediately upon mixing HA-BCN with the cross-linkers and the hydrogels reach their final stiffness within approximately 1 h. We compared this eECM with two commonly used eECMs: fibrin, a defined, natural, and pathophysiologically relevant eECM widely used in organ-on-a-chip systems; and Matrigel, an undefined yet vastly used eECM (Fig. 2*A*). We mixed HNSCCs/CAFs/HUVECs (HCHs) at a 1:3:1 ratio and cocultured them in HPVCL, Fibrin, or Matrigel at a total concentration of 15 × 10^6^ cells/mL (Fig. 2*A*) for 4 d. We then isolated HCHs from the eECM and assessed the effects of the eECMs on the global 3D HNSCC model gene expression by bulk RNA sequencing (N = 3 independent biological replicates per ECM; REFtoGEO). Bulk RNA sequencing revealed extensive ECM-dependent transcriptional remodeling (Log2Fc > |1| and adj. *P*-value < 0.05; Fibrin vs. HPVCL: 1,022 DEGs; Matrigel vs. HPVCL: 703 DEGs; Fibrin vs. Matrigel: 385 DEGs). Principal component analysis showed clear separation between the three eECM conditions, as HCHs cultured in HPVCL matrix, fibrin, and Matrigel each formed distinct transcriptional clusters with minimal overlap between biological replicates (Fig. 2*B*). Differential expression analysis further highlighted marked ECM-specific programs. Compared with fibrin and Matrigel, tumors cultured in the engineered HPVCL matrix showed increased expression of genes associated with squamous epithelial identity and clinically relevant HNSCC biomarkers, including *ERBB3*, *ALDH1A3*, *CD24*, *CEACAM6*, *LYPD3*, *TACSTD2*, *SERPINB3*, *SERPINB4*, and *MACC1*. In contrast, fibrin promoted an ECM-remodeling and mechanotransductive program characterized by increased expression of *CCN1, CCN2, SERPINE1, HAS2, TAGLN, TGFBI, THBS1*, and *FLT1*, consistent with activation of YAP/TGFβ-associated signaling pathways and matrix remodeling processes. Matrigel induced a distinct inflammatory and vascular signaling state marked by elevated *CCL2, CXCL8, IL1R1, IL18R1, TNFAIP6, FLT1, KDR, PDGFRA, FGF7*, and *EDNRB* (Fig. 2*C*). Exposure to different eECMs further caused significant alterations in the expression of genes involved in pathways key for HNSCC, such as ERBB, WNT, NOTCH, and YAP. HCHs cultured in HPVCL showed a strong ERBB/EGFR and WNT signature, driven by increased expression of *ERBB3*, *HBEGF*, *TGFA*, and *AXIN2, NKD1, LEF1, TCF7*, respectively. HCHs cultured in HPVCL also showed a milder NOTCH signature, driven by high expression of *NOTCH2*, *HES1*, *HEY1,* and *DLL4*. HCHs cultured in Matrigel showed a stronger NOTCH signature, while fibrin induced expression of YAP and TGFβ1-associated genes including *CCN1, CCN2, SERPINE1, AXL, ANKRD1, TGFB1, TGFB2*. To obtain an integrated view of ECM-dependent biological programs, pathway and signature activity scores were calculated from Hallmark gene sets and custom HNSCC-relevant signatures. The engineered HPVCL matrix was associated with increased activity of epithelial and cancer stem cell–associated programs, fibrin with proliferative and YAP-driven remodeling programs, and Matrigel with inflammatory-vascular signaling.

To determine whether the ECM-dependent transcriptional differences were accompanied by phenotypic changes, we examined HCH organization, proliferative activity, and susceptibility to cisplatin-induced apoptosis by immunofluorescence microscopy (Fig. 2*D*). HCHs cultured in the three eECMs adopted distinct multicellular architectures, with HPVCL promoting compact epithelial-like tumor masses, fibrin supporting a dispersed organization, and Matrigel displaying an intermediate phenotype with early vessel-like structures (Fig. 2*D*). These structural differences were accompanied by altered proliferative activity. Consistent with the enrichment of proliferation-associated signatures identified by RNA sequencing, HCHs cultured in fibrin showed the highest proportion of Ki67-positive cells, whereas HPVCL cultures showed the lowest proliferative activity and Matrigel displayed intermediate levels (Fig. 2*D*). Following cisplatin treatment, HCHs cultured in HPVCL displayed the highest proportion of cleaved caspase-3-positive cells, whereas the proportion of apoptotic cells was lower in fibrin and Matrigel cultures (Fig. 2*D*). To assess whether matrix-dependent drug availability contributed to these differences, we quantified cisplatin retention under cell-free conditions (*SI Appendix*, Fig. S2). All three matrices interacted with cisplatin, with HPVCL retaining more platinum than fibrin or Matrigel, suggesting that ECM-dependent therapeutic responses likely arise from both matrix–drug interactions and ECM-induced biological reprogramming.

### eECM Composition Induces Differential Self-Organization, Migration, Vasculature Invasion, and Response to Cisplatin of HNSCC at the Tumor–Vasculature Interface.

**Fig. 3.**
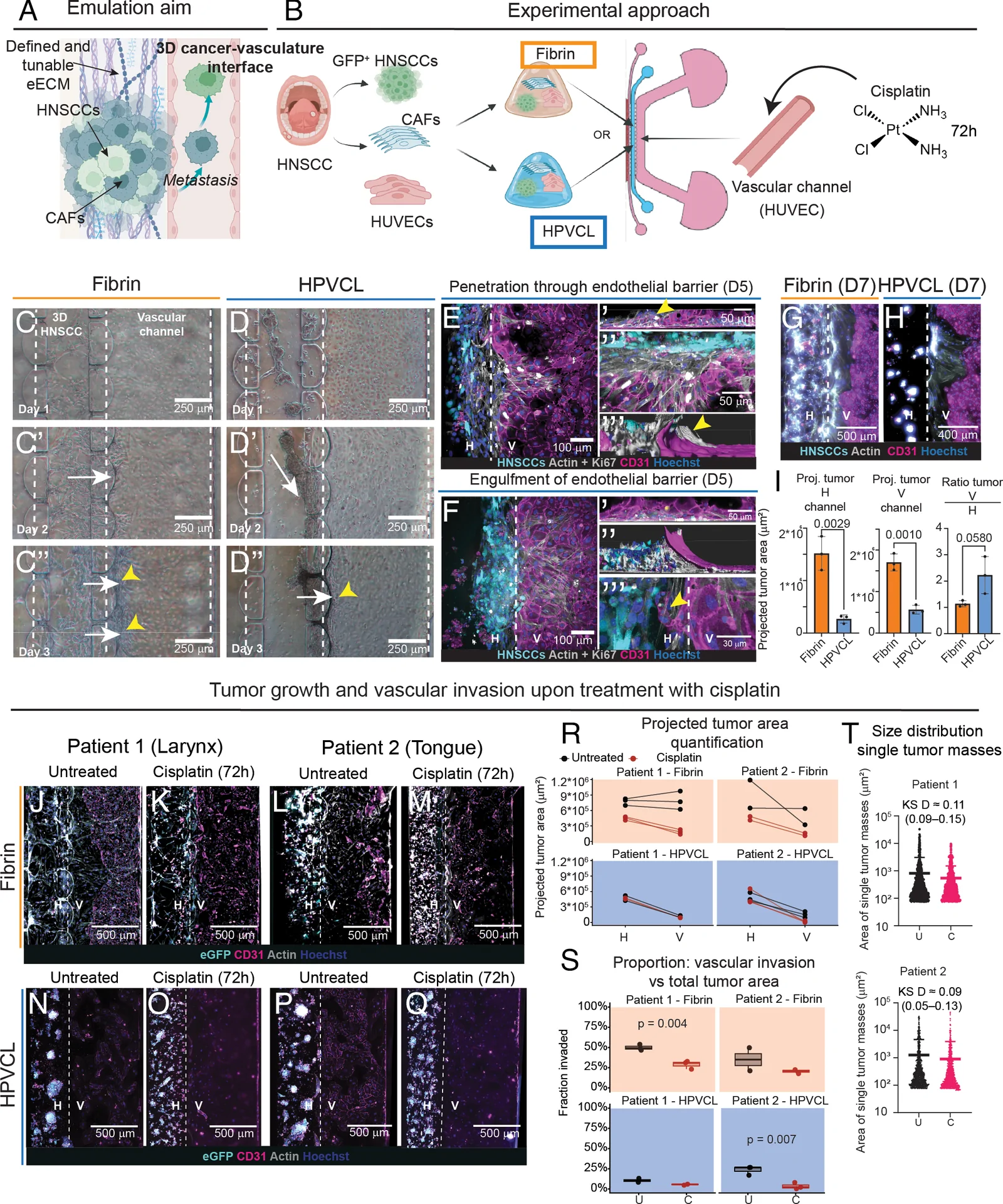
Emulation of the 3D HNSCC/ECM/vascular interface. (*A*) Emulation aim. (*B*) Experimental approach to coculture HCHs in different ECMs at the interface with a HUVEC-lined vascular channel. (*C* and *D*) Brightfield images showing ECM-dependent organization, migration, and vascular invasion of HCHs cultured in fibrin (*C*) or HPVCL (*D*) matrices. White arrows: migration direction; yellow arrowheads: representative sites of vascular invasion. (*E* and *F*) Immunofluorescent staining showing HCHs cultured in HPVCL eECM invading or engulfing the vascular channel through the endothelial barrier, day 4. Cyan: eGFP/HNSCCs; white: actin (CAFs) + Ki67; Magenta: CD31/HUVECs; blue: Hoechst33342/nuclei. H = HNSCC channel; V = vascular channel. (*E*, Top view and *E’*) view of the endothelial barrier and the invasion front from inside the vascular channel. The continuous endothelial barrier is interrupted by HCHs (yellow arrowhead). (*E”*) Detail of the invasion front. (*E’’’*) 3D rendering on the vascular invasion front. (*F*) Immunofluorescent staining showing HCHs cultured in HPVCL eECM engulfing the vascular channel, day 5. (*F*) Top-view, showing apparent invasion of the vascular channel. Yet, observation from within the vascular channel (*F’*) does not show any HCHs. 3D rendering (*F”*) highlights migration of HCHs, and mostly CAFs, below the endothelial layer. (*F”‘*) evidence of anastomosis or vascular sprouting from the main vascular channel into the 3D HNSCC. (*G* and *H*) Representative maximum intensity projections after 7 d of culture showing HCH organization in fibrin (*G*) and HPVCL (*H*) matrices. (*I*) Quantification of projected tumor area within the HNSCC (H) and vascular (V) compartments and the ratio of vascular to HNSCC tumor area after 7 d. (*J*–*Q*) Representative confocal images of fibrin- (*J*–*M*) and HPVCL-cultured (*N*–*Q*) HCHs from two independent patient-derived HNSCC lines following 96 h of culture, either with control medium (*J*, *L*, *N*, and *P*) or treated for 72 h with 3 µg/mL cisplatin via the vascular channel. (*R*) Compartment-based quantification of projected tumor area within the HNSCC (H) and vascular (V) compartments following cisplatin treatment. (*S*) Fraction invaded. Fraction of projected tumor area located within the vascular compartment (vascular area/total tumor area). Linear modeling showed significant effects of both extracellular matrix composition and cisplatin treatment on vascular invasion, whereas no significant hydrogel-by-treatment interaction was detected. (*T*) Size distributions of individual tumor masses following cisplatin treatment. Kolmogorov–Smirnov (KS) distances summarize differences between untreated and cisplatin-treated distributions. Statistical analysis. Projected tumor area was quantified separately within the HNSCC (H) and vascular (V) compartments. For vascular compartment area, linear modeling identified significant effects of extracellular matrix composition (*P* = 1.9 × 10^−5^) and cisplatin treatment (*P* = 2.2 × 10^−4^), whereas the hydrogel × treatment interaction was not significant (*P* = 0.43). Similarly, the fraction of tumor area located within the vascular compartment was significantly affected by hydrogel (*P* = 2.2 × 10^−5^) and cisplatin treatment (*P* = 7.6 × 10^−4^), with no significant interaction (*P* = 0.56). Significant hydrogel × treatment interactions were detected for projected tumor area within the HNSCC compartment (*P* = 0.0027) and for total projected tumor area (*P* = 6.5 × 10^−4^). Tumor size distributions (T) were compared using Kolmogorov–Smirnov (KS) distances, which are reported as effect sizes.

We increased the complexity of our modeling of the TME by integrating the fibrin-based and HPVCL-based 3D HNSCCs into our partial HNSCC-on-a-chip device, which allows the emulation of a 3D HNSCC–vascular interface, a common route for metastasis (33) (Fig. 3*A*). To this end, we resuspended HCHs either in HPVCL or fibrin, injected them in their dedicated chamber adjacent to the HUVEC-coated endothelialized channel, and monitored their behavior over time (Fig. 3*B*). Already after 24 h, cells displayed strong eECM-specific self-organization. While HCHs cultured in fibrin showed an elongated morphology and were distributed evenly across the matrix (Fig. 3*C*), HCHs cultured in HPVCL self-organized in multiple dense tumor masses (Fig. 3*D*). Within 2 d, HCHs cultured in fibrin migrated over multiple invasion fronts toward the vascular compartment and invaded it along the entire tumor–vasculature interface (Fig. 3 *C*–*C”*). HCHs cultured in HPVCL showed a radically different behavior: merged in tumor masses, they formed discrete invasion fronts that migrated as a collective unit through the eECM toward the vascular chamber, which they invaded by day three (Fig. 3 *D*–*D”* and *E*). By day 5, we observed multiple discrete invasion fronts into the vasculature (Fig. 3 *E’*–*E”’*). Overall, the vascular compartment exerted a strong attractive effect on the tumor mass: in several instances, we observed CAFs migrating around and below the endothelial cells, engulfing the vessel (Fig. 3 *F*–*F”’*). By day 7, most migration fronts invaded the vessel, causing the loss of the endothelial barrier between the vascular and the 3D HNSCC. However, not all HCHs invaded the vessel: we observed the persistence of nonproliferating, yet alive HCH-masses within the HPVCL-eECM well after the invasive fronts entered the vasculature (Fig. 3*H*). HCHs cultured in fibrin-eECM showed a radically different behavior, as they indiscriminately invaded the vascular chamber by day 7, creating a continuum with those remaining within the eECM. In this case, CAFs and HNSCC formed multiple, continuous invasion routes from the eECM to the vasculature, with no discernible morphological distinction (Fig. 3*G*). HCHs cultured in fibrin formed larger tumor masses within the HNSCC compartment and displayed higher invasion of the vascular compartment than HCHs cultured in HPVCL-eECM, resulting in a higher fraction of total tumor area located within the vascular compartment (Fig. 3*I*).

To investigate whether our partial HNSCC-on-a-chip device could be used to assess therapeutic responses in distinct tumor microenvironments, we treated HCHs cultured in fibrin- and HPVCL eECMs with cisplatin for 3 d and quantified tumor distribution between the HNSCC and vascular compartments (Fig. 3 *J*–*T*). We generated HCHs from HNSCCs isolated from two different patients. To enable a simple and scalable assessment of therapeutic responses within the vascularized chip, we quantified tumor area separately within the HNSCC and vascular compartments and quantified total tumor burden and tumor distribution between compartments. Using these compartment-based metrics, we found that cisplatin significantly reduced tumor area within the vascular compartment irrespective of the eECM (Fig. 3 *R* and *S*; see figure legend and *Materials and Methods* for statistical details of the analysis), indicating a general inhibitory effect on vascular invasion. No significant interaction between eECM and treatment was detected, suggesting that the anti-invasive effect of cisplatin was largely independent of the eECM. In contrast, analysis of tumor area within the original HNSCC compartment showed a marked reduction in tumor burden following cisplatin exposure for fibrin-cultured HCHs, while HPVCL-cultured HCHs were comparatively less affected (Fig. 3*R*). We further analyzed the size distributions of the distinct tumor masses that self-organized in the HPVCL chips. Only modest changes in tumor masses’ size distribution following cisplatin treatment were detected (Fig. 3*T*), indicating that treatment effects were captured more robustly by compartment-level measurements of tumor burden and vascular invasion. No significant patient effect was detected for any of the analyzed parameters (figure legend, *Materials and Methods*), indicating that the observed effects were primarily associated with extracellular matrix composition and cisplatin treatment. Together, these results demonstrate that the platform can resolve both microenvironment-dependent invasion phenotypes and extracellular matrix-dependent differences in therapeutic response.

### Generation of 3D hESC-Derived Human Bioengineered Trigeminal Ganglia (hbTG).

**Fig. 4.**
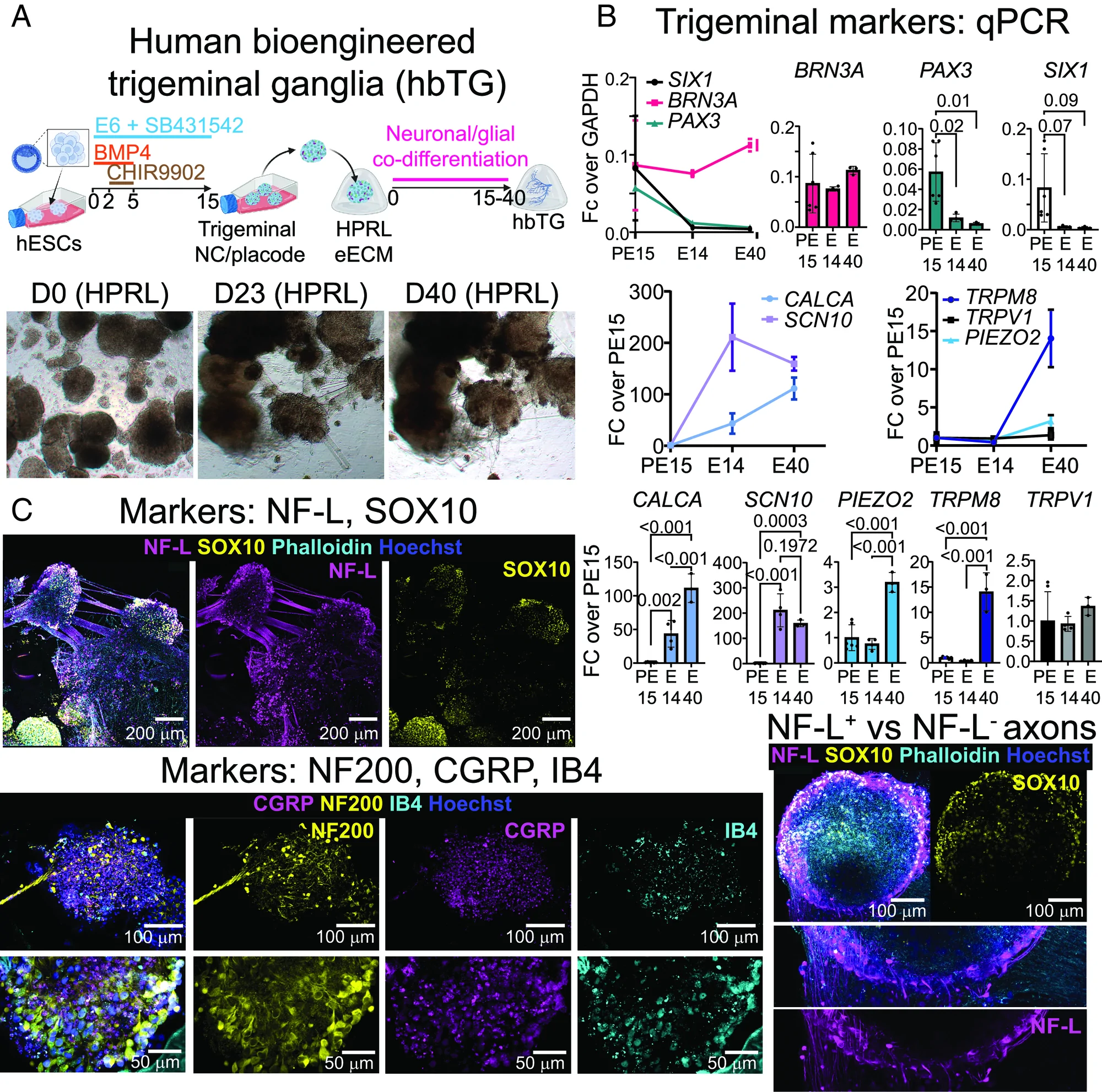
Generation of 3D hESC-derived bioengineered trigeminal ganglia (hbTG). (*A*) Schematic of the differentiation and bioengineering workflow. Representative brightfield images show ganglion formation and axonal outgrowth during maturation. (*B*) Characterization of trigeminal differentiation by quantitative RT-PCR. PreeECM day 15 (PE15): n = 6; eECM day 14 (E14): n = 4; eECM day 40 (E40): n = 3. One-way ANOVA with Tukey correction for multiple comparisons; exact *P* value (when *P* < 0.2) indicated on each comparison. (*C*) Immunofluorescence characterization of hbTG. N= 9 independent replicates per staining combination.

We then set out to generate 3D human bioengineered trigeminal ganglia (hbTG) in a fully defined and injectable 3D eECM. We adapted an existing protocol that generates diverse populations of trigeminal neurons, including neurons responsive to heat, cold, and inflammatory pain, as well as trigeminal glial cells from human pluripotent stem cells (Fig. 4*A*) (34). We first differentiated human embryonic stem cells (hESCs) into a mixed population of trigeminal neural crest and placode cells in 2D. We then detached the trigeminal precursors obtained, encapsulated them in an eECM composed of HA-BCN, PEG-Az8, cRGD, and Laminin 521 (HPRL), and cultured them in neuronal differentiation medium for up to 40 d (Fig. 4*A*). We obtained organization of trigeminal precursors into dense trigeminal ganglia-like structures that sprouted bundled axons into the eECM (Fig. 4*A*). hESC-derived bioengineered trigeminal ganglia (hbTG) expressed the trigeminal markers *PAX3* and *SIX1*, and the neuroblasts marker *BRN3A* (Fig. 4*B*). Expression of *PAX3* and *SIX1* was highest at the trigeminal precursor stage to then decrease during neuronal differentiation, in accordance with their expression profile during trigeminal development in vivo. Neuronal differentiation was accompanied by a strong increase in the expression of *CALCA* (marker for nociceptive neurons) (35) and of *SCN10A*, coding for the voltage-gated sodium channel Nav1.8 responsible for the transmission of action potentials (36). We further detected increased expression of *PIEZO2* (marker for mechanosensitive sensory neurons), *TRPM8* (marker for cold-responsive sensory neurons), and to a lesser extent *TRPV1* (marker for heat-responsive neurons) (37, 38). Immunofluorescence analysis showed formation of ganglion-like structures containing NF-L-positive neuronal networks surrounded by SOX10-positive glial cells, confirming the coexistence of neuronal and glial populations within hbTG (Fig. 4*C*). Axons extended as bundled nerve-like structures into the surrounding eECM, while SOX10-positive glial cells were detected both within the ganglia and along the extending axons. Immunostaining further revealed heterogeneous sensory neuronal populations, including NF200-positive neurons and CGRP-positive nociceptive neurons, whereas IB4 labeling identified an additional subset of sensory neurons, consistent with the emergence of multiple trigeminal sensory subtypes (Fig. 4*C*). The expression of nociceptive markers and SCN10A was accompanied by ATP-evoked calcium responses (Movie S1). Together, these data demonstrate the generation of multicellular human bioengineered trigeminal ganglia containing neuronal and glial populations and expressing markers associated with multiple sensory neuron subtypes, suitable for integration into the innervated HNSCC-on-a-chip platform.

### Trigeminal Innervation Reshapes HNSCC Organization and Compartmental Distribution Within the Tumor-on-a-Chip.

**Fig. 5.**
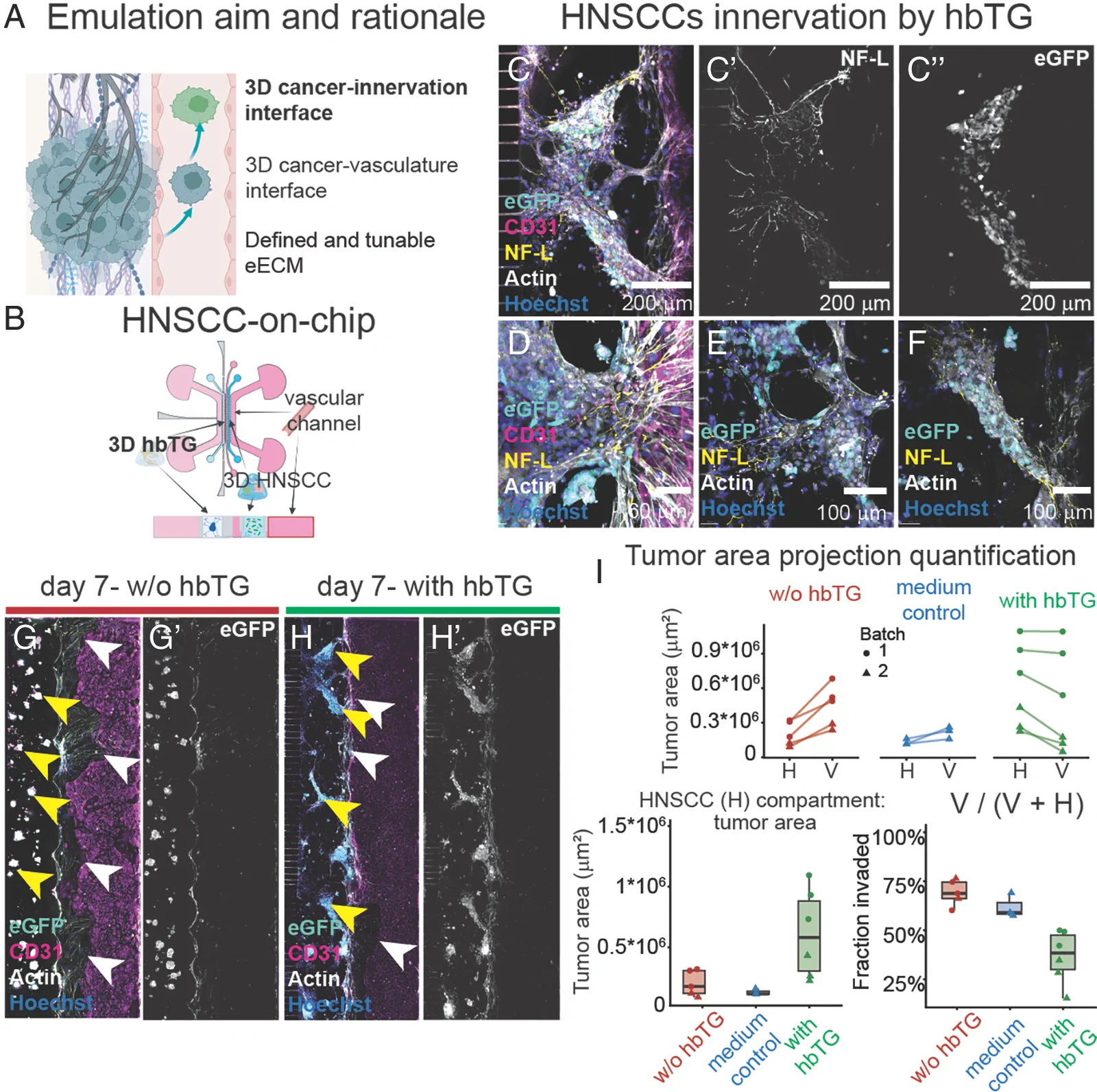
Complete HNSCC-on-a-chip: 3D trigeminal innervation-3D HNSCC–vasculature interface. (*A*) Emulation aim. (*B*) Experimental approach to coculture 3D HNSCC (HCHs) with a HUVEC-lined vascular channel and with hbTG. (*C*) Immunofluorescent staining showing extensive innervation of HCHs by hbTG at the interface with vasculature. Cyan: GFP/HNSCC; magenta: CD31/HUVEC; blue: Hoechst33342/nuclei; yellow: neurofilament light/NF-L; white: actin. (*C’*) neurofilament/NF-L channel. (*C”*) eGFP channel. (*D*) Higher magnification of C. (*E* and *F*) Immunofluorescent staining showing innervation of HCHs by hbTG on-a-chip. (*G*–*H’*) Immunofluorescent staining showing tumor area in presence (*H* and *H’*) or absence (*G* and *G’*) of trigeminal innervation after 7 d of culture on-a-chip. Yellow arrowheads: tumor masses. White arrowheads: vascular invasion fronts. (*I*) Compartment-based quantification of projected tumor area. *Upper* panel: projected tumor area within the HNSCC (H) and vascular (V) compartments for individual chips. *Lower* panels: projected tumor area within the HNSCC compartment and the fraction of total tumor area located within the vascular compartment. Linear modeling (batch corrected) identified significant effects of trigeminal innervation on projected tumor area within the HNSCC compartment (*P* = 0.0013) and on the fraction of tumor area located within the vascular compartment (*P* = 4.5 × 10^−4^). Data are shown as individual biological replicates; symbols indicate independent experimental batches. N = 6 chips for innervated HCHs, N = 6 for noninnervated HCHs, and N = 3 for gel/medium controls.

We assessed the effect of trigeminal innervation on the behavior of HCHs cultured in HPVCL on-chip. To this end, we injected 3D hbTGs and HCHs into the central adjacent chambers connected by microchannels, to allow axon projection from the hbTG to the HNSCC chamber. We further generated a vascular channel in the right-most channel, in direct contact with the HNSCC compartment (Fig. 5 *A* and *B*). All tumor structures in hbTG-containing chips were profusely innervated. Axons followed the borders of the tumor masses (Fig. 5 *C*–*F*) and preferentially grew along the tumor surface rather than within the eECM. Axons appeared to innervate both CAFs and HNSCCs without obvious preference. Notably, innervation was very abundant at many tumor–vascular interfaces, creating complex neurovascular-tumor bundles closely associated with vascular invasion (Fig. 5*D*).

Within 7 d, trigeminal innervation markedly altered the spatial organization of HCHs. While noninnervated HCHs formed small spheroid-like clusters (Fig. 5 *G* and *G’*), innervated cultures developed fewer and larger tumor structures, often spanning the entire breadth of the 3D HNSCC compartment (Fig. 5 *H* and *H’*). Consistent with these observations, compartment-based quantification showed a significant increase in tumor area within the original HNSCC compartment (Fig. 5*I*), while analysis of individual tumor masses confirmed a shift toward larger structures (KS D ≈ 0.34). Interestingly, although tumor cells were readily detected within the vascular compartment of both innervated and noninnervated cultures (Fig. 5 *G*–*H’*, white arrowheads), the presence of hbTGs was associated with a marked change in the relative distribution of tumor cells between compartments. Innervated cultures exhibited a significantly lower fraction of total tumor area located within the vascular compartment than either noninnervated condition (Fig. 5*I*). Importantly, exposure to hbTG culture medium alone did not affect this parameter, suggesting that the observed phenotype was associated with the presence of trigeminal innervation rather than with differences in culture conditions. Together, these findings establish the feasibility of integrating human trigeminal ganglia into an HNSCC-on-a-chip platform containing an endothelialized vascular compartment and show that compartment-based image analysis can detect changes in tumor organization and compartmental distribution associated with trigeminal innervation.

## Discussion

A central challenge in cancer biology is achieving a holistic and mechanistic understanding of the tumor microenvironment (TME), in which cancer cells interact with extracellular matrix (ECM), vasculature, nerves, and stromal populations under controlled physical and biochemical constraints. Traditional reductionist models have yielded invaluable insights into individual components of the tumor microenvironment, yet they fall short in capturing the behaviors that arise from their integration. In this context, organ-on-a-chip technologies offer human-specific platforms capable of bridging the gap between the fine mechanistic dissection enabled by in vitro models and in vivo complexity (3). The recent decision by regulatory agencies, including the US Food and Drug Administration, to allow, under defined circumstances, clinical testing pipelines that do not rely on animal experimentation further underscores the urgency and relevance of developing predictive, human-based preclinical models (39, 40).

In designing the present platform, we focused on three fundamental and broadly conserved interfaces that shape solid tumor behavior: tumor-extracellular matrix, tumor–vasculature, and tumor–innervation. Each of these elements is increasingly recognized as a driver of tumor progression, therapeutic response, and patient well-being (19, 41, 42). We thus devised a simple yet powerful design that allows simultaneous emulation and imaging of 3D neural, tumor, and vasculature compartments, in three adjacent channels. The central 3D HNSCC channel enables the study of the behavior of different cells composing HNSCC in an ECM of choice. The direct contact with the adjacent vascular channel enables the study of metastatization via vasculature, as well as the emulation of systemic delivery of drugs, therapeutic immune cells, and other therapeutic approaches. The asymmetric contact with vasculature could enable the study of hypoxia gradient formation (43). The direct contact between trigeminal axon terminals and the 3D HNSCC allows fine investigation of the effect of trigeminal innervation on HNSCC behavior. In a complete HNSCC-on-a-chip setup, the presence of a separated trigeminal chamber, connected to the 3D HNSCC chamber via microgrooves, allows imaging-based analysis of neuronal activity in response to tumor progression and therapeutic treatments, opening the possibility of studying HNSCC-associated pain-on-a-chip. While immune components are undeniably central to HNSCC and overall tumor biology, their integration into complex microfluidic systems poses additional challenges (44) and represents a logical next step, yet beyond the scope of the current work. Similarly, integration of a HNSCC–bone interface, which constitutes a clinically relevant HNSCC invasion route and was recently proposed in an innovative bone-on-a-chip device (22), will require further engineering of the platform.

The ECM plays a particularly critical role in HNSCC, where extensive clinical and experimental evidence links aberrant ECM composition and mechanics to invasion, treatment resistance, and poor prognosis (45–48). Most existing 3D HNSCC models rely on undefined matrices such as Matrigel® or Myogel, which suffer from batch-to-batch variability and limited tunability, or single-component ECMs, such as collagen and fibrin, which provide limited biochemical complexity (28, 49). These limitations hinder mechanistic interpretation and compromise translational relevance. By contrast, fully defined engineered ECMs enable precise control over stiffness, ligand composition, and degradability, parameters that are essential for dissecting how physical and biochemical cues regulate tumor–stroma interactions.

Our results show that ECM composition not only alters multicellular organization but also profoundly reshapes the transcriptional state of engineered HNSCC microtissues. RNA sequencing revealed ECM-dependent programs associated with mechanotransduction, inflammatory signaling, and clinically relevant oncogenic pathways, demonstrating that matrix selection is itself a major determinant of the biology captured by engineered tumor models. These distinct transcriptional responses likely emerge from the combined influence of ECM composition and biophysical properties, which together define the cellular microenvironment and were not independently varied in the present study. In isolated 3D cultures, HCHs embedded in HPVCL displayed greater cisplatin-induced apoptosis than those cultured in fibrin or Matrigel. Based on mesh size alone, fibrin ECMs should allow easier diffusion of cisplatin than HPVCL, as the pore size is in the range of micrometers for fibrin ECMs (50) and of nanometers for HPVCL eECMs (32). Together with our ICP-MS measurements, these observations suggest that ECM-dependent therapeutic responses cannot be explained by passive drug diffusion alone, but instead arise from the combined effects of matrix–drug interactions and ECM-induced changes in the multicellular state. Vascularization is a second essential dimension of tumor pathophysiology, providing trophic support, waste removal, and metastatic dissemination. By culturing 3D HCHs constructs adjacent to an endothelial cells-lined vascular channel, we observed rapid, robust, directional migration of tumor cells toward the vascular compartment. Whether this behavior is driven primarily by gradients of trophic support or endothelial cells-derived signaling cues remains to be determined. When cultured adjacent to an endothelialized vascular channel, 3D HCHs showed radically different self-organization and migratory patterns depending on the ECM chosen. 3D HCHs cultured in HPVCL self-organized in discrete tumor masses, as observed when they were cultured in simple 3D cultures, and then migrated collectively toward the vascular channel. On the other hand, 3D HCHs cultured in fibrin migrated on several independent paths from the ECM to the vasculature. When cultured for 7 d, HCHs embedded in HPVCL matrix displayed a pronounced dimorphism, with distinct cellular subpopulations emerging near and distant from the vasculature, a phenomenon absent in fibrin-based matrices. This observation reinforces the possibility that ECM composition may regulate the balance and spatial distribution between quiescent, invasive, and proliferative tumor cell states, a hypothesis with important implications for metastasis and therapy resistance (51). The response to cisplatin in chips containing the endothelialized vascular compartment was attenuated compared with isolated 3D tumor cultures, a finding consistent with emerging literature in vascularized tumor models. Interestingly, the effect of ECM composition on cisplatin response differed between the isolated 3D cultures and on chip. Whereas HPVCL-cultures showed greater cisplatin-induced apoptosis in isolated domes, fibrin cultures exhibited a larger reduction in tumor burden in the chip. These findings can be reflective of the distinct biological processes captured by the two systems. The isolated dome assay measures acute apoptosis following relatively homogeneous drug exposure, whereas the chip integrates delivery via an endothelialized channel, tumor architecture, proliferation, migration, and invasion. Consequently, ECM-dependent therapeutic responses emerge not only from intrinsic cellular sensitivity to cisplatin but also from the way the microenvironment shapes drug accessibility and tumor behavior. While this effect cannot be readily attributed to impaired drug diffusion, as substantially larger molecules readily diffuse from the vascular channel through the 3D ECMs, the delivery purely from the vascular channel potentially increases the barrier effect exerted by the ECM. Endothelial cells themselves, via endothelial cells-mediated drug sequestration, metabolic modulation, or pro-survival signaling, may further contribute to the observed protection (52, 53). Addressing this question will require future enrichment of the model, for example by incorporating more physiologically complete vessel walls, including pericytes, smooth muscle cells, and tumor-derived endothelial-like cells.

An advantage of the present study is the use of simple compartment-resolved tumor area measurements as phenotypic readouts of tumor progression and therapeutic response within a vascularized tumor-on-a-chip platform, as they provide a simple phenotypic readout that captures both tumor growth and invasive behavior. While this approach does not replace high-content molecular analyses, it offers a rapid and scalable strategy for comparing microenvironment-dependent phenotypes and therapeutic responses.

A distinctive feature of the present platform is the incorporation of sensory innervation. Growing evidence shows that neural activity is an active regulator of tumor growth and invasion, particularly in HNSCC (17, 54), where trigeminal innervation also underlies cancer-associated pain, a major clinical burden and a frequent dose-limiting side effect of cisplatin (55). Here, we used human embryonic stem cells to generate 3D bioengineered trigeminal ganglia (hbTG) that robustly innervate 3D HCHs. Building on an established hiPSC-derived trigeminal neuron differentiation protocol, which demonstrated codifferentiation of action potential-competent and diverse trigeminal neuronal populations (34), we adapted the system to a 3D defined ECM environment. The resulting hbTGs showed coexistence of SOX10-expressing glial cells and NF200-, CGRP-, and IB4-labeled neuronal populations, supporting the emergence of heterogeneous trigeminal sensory neuron-like identities in 3D, and in agreement with the original publication. We decided to proceed via predifferentiation in 2D, followed by encapsulation and maturation in an injectable and fully defined 3D eECM, rather than using well-established sensory organoids (56), to minimize damage to neuronal structures when injecting the 3D hbTG in the chip. An unexpected advantage consisted in the abundant sprouting and bundling of axons from the self-organized hbTG through the eECM toward the edges of the 3D construct, reminiscent of in vivo patterns of axon sprouting and nerve bundling in the trigeminal complex (57). Other designs have been proposed that allow insertion of ganglia and larger organoids within organ-on-a-chip devices without injection (58, 59). Yet, we believe that our “fully-injectable-ganglia” strategy provides a valuable, complementary approach that can significantly expand the possibilities to integrate complex innervation in a variety of new and existing organ-on-a-chip designs. The use of fully defined eECMs furthermore opens the possibility to systematically assess whether and how eECM properties affect the establishment of neuronal and glial cell diversity. Notably, trigeminal innervation was associated with altered tumor organization and compartmental distribution. Innervated cultures exhibited increased tumor accumulation within the primary tumor compartment and a reduced proportion of tumor area located within the vascular compartment. Although the limited sample size precludes strong conclusions regarding the underlying biological mechanisms, these observations are consistent with the notion that innervation can influence tumor behavior through biochemical, electrical, and physical interactions. Potential mechanisms may include the release of signaling molecules from axon terminals, bidirectional electrical and biochemical signaling, as well as axons serving as physical scaffolds, paralleling observations from in vivo studies that support complex effects of innervation on HNSCC progression and metastatization (54, 60, 61). ATP-evoked calcium transients demonstrate that the bioengineered trigeminal ganglia are functionally responsive to stimulation. However, these measurements do not prove mature electrophysiological properties, whose characterization will require future validation using electrophysiological approaches. Beyond tumor biology, this innervated platform opens the door to future investigations of chemotherapy–innervation interactions, enabling studies of how sensory innervation influences therapeutic responses, how chemotherapeutic agents affect neuronal viability and network integrity, and how these processes contribute to chemotherapy-induced neuropathy. Our platform provides an opportunity to bridge clinical observations with mechanistic investigations in a fully human microphysiological system while adhering to the principles of replacement, reduction, and refinement (3R) of animal experiments.

In summary, we developed a fully defined, innervated, and vascularized HNSCC-on-a-chip platform that captures key structural and functional determinants of HNSCC pathophysiology. More broadly, this approach provides a versatile basis for investigating solid tumor responses to therapy, drug transport across ECM and vascular barriers, and the interactions between complex 3D cancer models and cancer-specific innervation. We anticipate that continued refinement of this platform will contribute to more predictive preclinical testing and to the rational design of therapies that address not only tumor control, but also patients’ quality of life.

## Materials and Methods

### Cell Culture.

The study protocol, including the use of HNSCC cell lines derived from primary tumor biopsies obtained at the Department of Otorhinolaryngology–Head and Neck Surgery, Linköping University Hospital (Sweden), was approved by the Regional Ethical Review Board in Linköping (approval no. 03-537). Written informed consent was obtained from all patients, and all samples were deidentified prior to use. All procedures were conducted in accordance with applicable ethical guidelines and regulations. Tumor cell lines (HNSCCs) and CAFs were established from pretreatment HNSCC biopsies as described in the original publication (62). HNSCCs and CAFs were cultured in DMEM/F12 (Cat.#11320033, ThermoFisher Scientific) supplemented with 10% Fetal Bovine Serum (FBS; Cat.#A5256701; ThermoFisher Scientific), and 100 U/mL of penicillin/streptomycin (Cat.#15140122, ThermoFisher Scientific). When confluent, cells were passaged by incubation in trypsin/EDTA 0.25% (Cat.#25200056, ThermoFisher Scientific) followed by splitting at the desired concentration, without centrifugation steps. Human umbilical cord vein endothelial cells (HUVECs; Cat.#C-12200, Sigma-Aldrich) were cultured in endothelial growth medium (EGM-2; Cat.#C-22211, Sigma Aldrich) supplemented with EGM-2 supplement mix (Cat.#C-39216, Sigma-Aldrich) and 100 U/mL of penicillin/streptomycin on collagen I (PureCol® Type I Collagen; Cat.#5074-35ML, CELLInk)-coated flasks. HUVECs were detached by incubation in Tryple Express (Cat.#12604021, ThermoFisher Scientific) and plated at the desired concentrations. All experiments were performed using HUVECs between passage IV-VIII. MESP1mCherry/w-NKX2-5eGFP/w human embryonic stem cells (63) were kindly provided by Claudio Cantù (Linköping University). hESCs were cultured in Essential 8 medium (Cat.#A1517001, ThermoFisher Scientific) on vitronectin-coated (0.1 mg/mL in PBS; Cat.#A14700, ThermoFisher Scientific) plastic tissue flasks. When locally confluent, hESCs were detached via incubation in 0.5 mM EDTA, diluted in Essential 8, and split 1:3 to 1:12.

### Hydrogels.

#### Hyaluronic acid/PEG-based hydrogels.

The formulations of all the eECMs used in this work are reported in *SI Appendix*, Table S1 and described in detail in *SI Appendix*. Hyaluronic acid-based hydrogels were prepared by mixing HA-BCN and PEG-Az8 at a 4:1 molar ratio. The total polymer stock concentrations used were 1% (w/v%), 1.5%, or 2%, with the volumes adjusted accordingly to maintain the desired ratios. Hydrogels were supplemented with the desired bioactive functionalization (64). Cells or trigeminal placodes were resuspended in HA-BCN at the desired concentration; the additional hydrogel components were then added to the HA-BCN-cell suspension, with Az-bearing crosslinking agents added at the end. Samples were incubated at 37 °C to allow hydrogel crosslinking before culture medium addition, with crosslinking times defined according to the specific hydrogel formulation and experimental application, as detailed in the corresponding sections.

#### Fibrin-based hydrogels.

Human fibrinogen (100 mg/mL in 0.9% NaCl; Cat.#F3879-250MG, Sigma-Aldrich) and thrombin (100 U/mL in 40 mM CaCl₂; Cat.#T6884-100UN, Sigma-Aldrich) stock solutions were diluted to final working concentrations of 40 mg/mL and 10 U/mL, respectively. With fibrinogen and thrombin on-ice, cells were resuspended in thrombin. The two components were mixed at a 1:1 volume ratio immediately before use to initiate fibrin polymerization. Loaded fibrin hydrogels were allowed to solidify for 10 min at 37 °C in the incubator, before adding culture medium.

### HNSCCs-CAFs-HUVECs Dome Cultures.

HNSCCs, CAFs, and HUVECs were mixed at a 1:3:1 ratio, encapsulated in HPVCL, fibrin, or Matrigel at 15 × 10^6^ cells/mL, and cultured as 3D domes for 3 d. For assessment of cisplatin effect, dome cultures were treated with 3 μg/mL cisplatin from day 1 to day 3 with daily medium replacement. Matrix-dependent cisplatin retention was quantified by ICP-MS after incubation of cell-free hydrogels with cisplatin. All imaging data were analyzed with Fiji/ImageJ (65). Detailed hydrogel formulations, preparation procedures, and analysis description are provided in *SI Appendix*, Table S1.

### hESCs-Derived 3D Trigeminal Ganglia.

Human embryonic stem cells (hESCs) were differentiated into trigeminal ganglia by adapting a published protocol (34). Trigeminal placode progenitors were generated in two-dimensional culture for 14 d and subsequently encapsulated within the HA-PEG-cRGD-laminin (HPRL) hydrogel, where differentiation and maturation continued in three-dimensional culture for up to 40 d to generate human bioengineered trigeminal ganglia (hbTG). Complete protocols, culture conditions, and imaging procedures are provided in the *SI Appendix*.

### Real Time qPCR (RT-qPCR).

3D eECMs containing hbTG were disrupted by incubation with TRIzol (Cat.#15-596-018, ThermoFisher Scientific) combined with manual mechanical disaggregation, and RNA was extracted using the Direct-zol RNA purification kit (Cat.#R2052, Zymo Research). RNA was reverse-transcribed into cDNA with High-Capacity RNA-to-cDNA Kit (Applied Biosystems, MA), using a LifeTouch thermal cycler (Bioer Technology, Zhejiang, China). RT-qPCR (Primers: *SI Appendix*, Table S2) was performed on a Light-Cycler 480 PCR System (Roche) and targets amplified using PowerUp SYBR Green Master Mix (Cat.#A25777, ThermoFisher Scientific; thermal cycling conditions: 95 °C 10 min, 40 cycles at 95 °C for 15 s, 55 °C for 10s, and 60 °C for 30 s). Data were normalized to the geometric mean of the reference genes. Relative expression levels were calculated using the ΔCt method or the comparative Ct (ΔΔCt) method.

### RNA Sequencing.

HCH cultures consisting of HNSCC cells, cancer-associated fibroblasts (CAFs), and endothelial cells were cultured in HPVCL, fibrin, or Matrigel matrices and harvested for bulk RNA sequencing (n = 3 independent biological replicates per condition). RNA was isolated from intact multicellular constructs without prior separation of individual cellular compartments. Libraries were generated and sequenced by Plasmidsaurus (USA) using a 3′ UMI-based RNA sequencing workflow. Sequencing reads were quality controlled, filtered, aligned to the reference genome, deduplicated using unique molecular identifiers, and quantified at the gene level using a standard RNA-seq analysis pipeline. Differential expression analyses were performed in R using edgeR following filtering of lowly expressed genes and TMM normalization. Analyses are described in detail in the *SI Appendix*.

### Microfluidic Devices Design and Fabrication.

The microfluidic device layout was designed using CAD software (AutoCAD®, AutoDesk Inc.). It consists of two main compartments for 3D cell-laden hydrogel cultures, one dedicated to the trigeminal compartment (hbTG compartment) and the other to the target tissue, represented by the 3D HNSCC model (HNSCC compartment). The hbTG compartment is a single-channel chamber with an inlet (⌀ = 1 mm) for 3D hbTG gel injection and an external channel with two reservoirs (⌀ = 5 mm) for medium supply. Similarly, the HNSCC compartment is a single-channel chamber, featuring gel injection ports (⌀ = 1 mm) to accommodate HNSCCs, CAFs, and HUVECs resuspended in the eECM of choice. The vascular compartment includes two reservoirs (⌀ = 5 mm) that serve as inlets for HUVECs seeding. The pillars in the trigeminal compartment have a trapezoidal cross-section, with a maximum length of 200 μm and a minimum interpillar distance of 50 μm. In contrast, the target tissue compartment features moon-shaped pillars, measuring 305.1 μm in length with 30.5 μm spacings between them. The pillars’ geometry, size, and spacing were specifically designed to retain the hydrogel upon injection. Detailed description of devices’ fabrication and characterization can be found in the *SI Appendix*.

### Culture of HNSCC-on-a-Chip.

#### Vascularized HNSCCs-on-chip.

HCHs gel suspensions were prepared as described above. 3 µL of this suspension were carefully injected into the 3D HNSCC channel and incubated at 37 °C and 5% CO₂ until complete gelation occurred (10 min). The vascular channel was coated with 0.1 mg/mL fibronectin (Sigma Aldrich) for 45 min at 37 °C. After washing the vascular channel with PBS, the reservoirs were filled with complete EGM-2 medium. Once a stable hydrostatic flow was established, 5 µL of HUVEC suspension at a density of 15 × 10^6^ cells/mL was injected into each vascular channel. When endothelial coverage was incomplete, an additional HUVEC seeding step was performed. The devices were then placed in a Petri dish (⌀ = 5 cm) in the incubator. From Day 1 on, cells were cultured in a 1:1 mixture of supplemented EGM-2/DMEM-F12, with daily medium changes. For chips loaded with fibrin hydrogels, aminocaproic acid (ACA, 2 mg/mL; Cat.#A7824, ThermoFisher Scientific) was added to the culture medium from Day 1 to prevent fibrin degradation.

#### Innervated and vascularized HNSCCs-on-a-chip.

To generate an innervated and vascularized HNSCC-on-a-chip model, hbTG in HPRL and HNSCC/CAF/HUVEC in HPVCL (*SI Appendix*, Table S1) were simultaneously injected into the two adjacent dedicated channels (Fig. 1). For on-chip integration, day-14 trigeminal placode progenitors were encapsulated in the HPRL hydrogel and injected into the 3D hbTG channel, where maturation occurred under complete Neurobasal medium supplied through the adjacent hbTG medium channel. Immediately after gelation (45 min for HPRL-hbTG and 30 min for the HPVCL-HCH), the “vascular channel” was treated as described above, while the “hbTG medium channel” was filled with E6 medium. On Day 1, the medium in the “hbTG medium channel” was replaced with complete Neurobasal medium. For the “medium control” condition, the tumor and innervation channels were injected simultaneously, with the innervation channel receiving HPRL gel only, without embedded ganglia. Following gelation of both compartments, E6 medium was added to the reservoirs on the “hbTG medium channel”, whereas the “vascular channel” was handled as described above. From day 1 onward, E6 medium was replaced with complete Neurobasal medium until the end of the experiment.

#### Cisplatin treatment on-chip.

Following chip seeding, the partial vascularized HNSCC-on-a-chip models were maintained in supplemented EGM-2 without cisplatin on Day 0. On Day 1, the medium was replaced with the 1:1 supplemented EGM-2/DMEM-F12 mixture supplemented with cisplatin at a final concentration of 3 µg/mL. The cisplatin-containing medium was replenished daily and maintained until Day 4, corresponding to the end of the experiment. Drug delivery to the 3D HNSCC compartment occurred through the adjacent vascular channel under passive hydrostatic flow. Untreated control chips were maintained under the same daily medium replacement schedule using culture medium without cisplatin.

#### Immunofluorescence staining.

For on-chip staining, all solutions were introduced through the device reservoirs and allowed to fill the corresponding microfluidic channels by passive flow. Samples were fixed with 4% formaldehyde (Thermo Fisher) for 15 min and permeabilized with Triton X-100 (0.1% in PBS) for 10 min at RT. Nonspecific binding sites were blocked with Bovine serum Albumin [2% (w/v) BSA; Thermo Fisher] in PBS for 1 h at RT. Chips or domes were then incubated with primary antibodies (*SI Appendix*, Table S3) overnight at 4 °C, then with the appropriate secondary antibodies (*SI Appendix*, Table S3) overnight at 4 °C (chips) or for 2 h at room temperature (domes), counterstained with Phalloidin Alexa Fluor™ 488 (1:1,000 dilution; Cat.#A12379; ThermoScientific) and for 30 min with Hoechst 33342 (1:10,000; Cat.#1932420, ThermoFisher Scientific). hbTG domes were mounted between the slide and a coverslip using a thin silicon spacer to preserve sample thickness and avoid compression of the 3D hydrogel constructs. Samples were imaged with a Leica Stellaris 5 CLSM and with a Leica DMi8 Widefield Microscope. Finally, images were analyzed with Fiji/ImageJ (65) and Imaris Viewer (Oxford Instruments). For tumor size analysis, see *SI Appendix*.

## Supplementary Material

Appendix 01 (PDF)

Dataset S01 (CSV)

Dataset S02 (CSV)

Dataset S03 (CSV)

Dataset S04 (TXT)

Movie S1. **ATP-evoked calcium responses in human bioengineered trigeminal ganglia (hbTG)**. Live calcium imaging of day-40 hbTG expressing the genetically encoded calcium indicator jGCaMP8f following stimulation with 1 mM ATP. hbTG were transduced with AAV9-CAG-jGCaMP8f-WPRE on day 34 of differentiation in the engineered extracellular matrix.

## Data Availability

RNA sequencing data (REFtoGEO) have been deposited in Gene Expression Omnibus (GSE336833) (66). All other data are included in the article and/or supporting information.

## References

[1] I. W. Mak, N. Evaniew, M. Ghert, Lost in translation: Animal models and clinical trials in cancer treatment. Am. J. Transl. Res. **6**, 114–118 (2014). PMC3902221 24489990

[2] D. E. Ingber, Human organs-on-chips for disease modelling, drug development and personalized medicine. Nat. Rev. Genet. **23**, 467–491 (2022). 10.1038/s41576-022-00466-9 PMC8951665 35338360

[3] C. M. Leung , A guide to the organ-on-a-chip. Nat. Rev. Methods Primers **2**, 33 (2022).

[4] S. N. Bhatia, D. E. Ingber, Microfluidic organs-on-chips. Nat. Biotechnol. **32**, 760–772 (2014). 10.1038/nbt.2989 25093883

[5] S. Jalili-Firoozinezhad, C. C. Miranda, J. M. S. Cabral, Modeling the human body on microfluidic chips. Trends Biotechnol. **39**, 838–852 (2021). 10.1016/j.tibtech.2021.01.004 33581889

[6] D. Pulte, H. Brenner, Changes in survival in head and neck cancers in the late 20th and early 21st century: A period analysis. Oncologist **15**, 994–1001 (2010). 10.1634/theoncologist.2009-0289 PMC3228039 20798198

[7] Q. Li, Y. Tie, A. Alu, X. Ma, H. Shi, Targeted therapy for head and neck cancer: Signaling pathways and clinical studies. Signal Transduct. Target. Ther. **8**, 31 (2023). 10.1038/s41392-022-01297-0 PMC9842704 36646686

[8] S. B. Hobday , Outcomes among patients with mucosal head and neck squamous cell carcinoma treated with checkpoint inhibitors. JAMA Otolaryngol. Head Neck Surg. **148**, 918–926 (2022). 10.1001/jamaoto.2022.2284 PMC9389441 35980666

[9] M. G. da Costa Sousa, , Engineering models of head and neck and oral cancers on-a-chip. Biomicrofluidics **18**, 021502 (2024). 10.1063/5.0186722 PMC10919958 38464668

[10] J. H. Choi , Single-cell transcriptome profiling of the stepwise progression of head and neck cancer. Nat. Commun. **14**, 1055 (2023). 10.1038/s41467-023-36691-x PMC9958029 36828832

[11] R. Kalluri, M. Zeisberg, Fibroblasts in cancer. Nat. Rev. Cancer **6**, 392–401 (2006). 10.1038/nrc1877 16572188

[12] A. Östman, M. Augsten, Cancer-associated fibroblasts and tumor growth–Bystanders turning into key players. Curr. Opin. Genet. Dev. **19**, 67–73 (2009). 10.1016/j.gde.2009.01.003 19211240

[13] O. D. Wever, P. Demetter, M. Mareel, M. Bracke, Stromal myofibroblasts are drivers of invasive cancer growth. Int. J. Cancer **123**, 2229–2238 (2008). 10.1002/ijc.23925 18777559

[14] J. Overgaard, Hypoxic radiosensitization: Adored and ignored. J. Clin. Oncol. **25**, 4066–4074 (2007). 10.1200/JCO.2007.12.7878 17827455

[15] J. Fares, M. Y. Fares, H. H. Khachfe, H. A. Salhab, Y. Fares, Molecular principles of metastasis: A hallmark of cancer revisited. Signal Transduct. Target. Ther. **5**, 28 (2020). 10.1038/s41392-020-0134-x PMC7067809 32296047

[16] A. C. Restaino , Functional neuronal circuits promote disease progression in cancer. Sci. Adv. **9**, 1–18 (2023). 10.1126/sciadv.ade4443 PMC10171812 37163587

[17] M. Amit , Loss of p53 drives neuron reprogramming in head and neck cancer. Nature **578**, 449–454 (2020). 10.1038/s41586-020-1996-3 PMC9723538 32051587

[18] C. C. Reyes-Gibby , Survival patterns in squamous cell carcinoma of the head and neck: Pain as an independent prognostic factor for survival. J. Pain **15**, 1015–1022 (2014). 10.1016/j.jpain.2014.07.003 PMC4418559 25043982

[19] Z. Yuan , Extracellular matrix remodeling in tumor progression and immune escape: From mechanisms to treatments. Mol. Cancer **22**, 48 (2023). 10.1186/s12943-023-01744-8 PMC10007858 36906534

[20] Z. Mai, Y. Lin, P. Lin, X. Zhao, L. Cui, Modulating extracellular matrix stiffness: A strategic approach to boost cancer immunotherapy. Cell Death Dis. **15**, 37 (2024). 10.1038/s41419-024-06697-4 PMC11063215 38693104

[21] I. Klabukov , Tumor-Associated extracellular matrix obstacles for CAR-T cell therapy: Approaches to overcoming. Curr. Oncol. **32**, 79 (2025). 10.3390/curroncol32020079 PMC11854105 39996879

[22] M. G. da Costa Sousa , Endogenous osteocyte-osteoclast signaling enables growth factor-free bone remodeling, drug response, and cancer invasion in a nanoscale calcified bone-on-a-chip model. bioRxiv [Preprint] (2025). http://doi.org10.64898/2025.12.08.693047 (Accessed 13 January 2026).

[23] S. M. Hattersley , A microfluidic system for testing the responses of head and neck squamous cell carcinoma tissue biopsies to treatment with chemotherapy drugs. Ann. Biomed. Eng. **40**, 1277–1288 (2012). 10.1007/s10439-011-0428-9 21997391

[24] R. Cheah , Measuring the response of human head and neck squamous cell carcinoma to irradiation in a microfluidic model allowing customized therapy. Int. J. Oncol. **51**, 1227–1238 (2017). 10.3892/ijo.2017.4118 28902347

[25] S. D. Carr, V. L. Green, N. D. Stafford, J. Greenman, Analysis of radiation-induced cell death in head and neck squamous cell carcinoma and rat liver maintained in microfluidic devices. Otolaryngol. Head Neck Surg. **150**, 73–80 (2014). 10.1177/0194599813507427 24098006

[26] R. Bower , Maintenance of head and neck tumor on-chip: Gateway to personalized treatment? Future Sci. **3**, FSO174 (2017). 10.4155/fsoa-2016-0089 PMC5481812 28670466

[27] R. Kennedy , A patient tumour-on-a-chip system for personalised investigation of radiotherapy based treatment regimens. Sci. Rep. **9**, 6327 (2019). 10.1038/s41598-019-42745-2 PMC6474873 31004114

[28] A. Al-Samadi , In vitro humanized 3D microfluidic chip for testing personalized immunotherapeutics for head and neck cancer patients. Exp. Cell Res. **383**, 111508 (2019). 10.1016/j.yexcr.2019.111508 31356815

[29] V. Green , Head and neck squamous cell carcinoma biopsies maintained ex vivo on a perfusion device show gene changes with time and clinically relevant doses of irradiation. Cancers **15**, 4575 (2023). 10.3390/cancers15184575 PMC10527562 37760543

[30] A. Scemama , Development of an in vitro microfluidic model to study the role of microenvironmental cells in oral cancer metastasis. F1000Res. **12**, 439 (2024). 10.12688/f1000research.131810.2 PMC10904955 38434654

[31] M. K. Heenkenda , Tissue-stiffness–driven platelet activation emerges as a potential target for breast cancer prevention and treatment. Cell Commun. Signal. **24**, 429 (2026). 10.1186/s12964-026-03138-x PMC13445922 42563161

[32] A. Abrahamsson , Increased matrix stiffness enhances pro-tumorigenic traits in a physiologically relevant breast tissue- monocyte 3D model. Acta Biomater. **178**, 160–169 (2024). 10.1016/j.actbio.2024.02.021 38382828

[33] N. S. Mastronikolis , Insights into metastatic roadmap of head and neck cancer squamous cell carcinoma based on clinical, histopathological and molecular profiles. Mol. Biol. Rep. **51**, 597 (2024). 10.1007/s11033-024-09476-8 PMC11058607 38683372

[34] B. Zimmer , Human iPSC-derived trigeminal neurons lack constitutive TLR3-dependent immunity that protects cortical neurons from HSV-1 infection. Proc. Natl. Acad. Sci. U.S.A. **115**, E8775–E8782 (2018). 10.1073/pnas.1809853115 PMC6140487 30154162

[35] C. G. Perez-Pacheco , Nerve types and classification of neurons innervating oral cancer. Clin. Cancer Res. **31**, 2741–2755 (2025). 10.1158/1078-0432.CCR-24-2375 PMC12281553 40310470

[36] M. Alsaloum , Voltage-gated sodium channels in excitable cells as drug targets. Nat. Rev. Drug Discov. **24**, 358–378 (2025). 10.1038/s41573-024-01108-x PMC12379884 39901031

[37] S. A. Bhuiyan , Harmonized cross-species cell atlases of trigeminal and dorsal root ganglia. Sci. Adv. **10**, eadj9173 (2024). 10.1126/sciadv.adj9173 PMC11804847 38905344

[38] L. Yang , Human and mouse trigeminal ganglia cell atlas implicates multiple cell types in migraine. Neuron **110**, 1806–1821.e8 (2022). 10.1016/j.neuron.2022.03.003 PMC9338779 35349784

[39] M. Wadman, FDA no longer has to require animal testing for new drugs. Science **379**, 127–128 (2023). 10.1126/science.adg6276 36634170

[40] Food, D. Administration (FDA), “Roadmap to reducing animal testing in preclinical safety studies” (2025). 10.1002/cpdd.70046 41766281

[41] X. Li , Targeting tumor innervation: Premises, promises, and challenges. Cell Death Discov. **8**, 1–12 (2022). 10.1038/s41420-022-00930-9 PMC8956600 35338118

[42] V. S. Shirure, C. C. W. Hughes, S. C. George, Engineering vascularized organoid-on-a-chip models. Annu. Rev. Biomed. Eng. **23**, 141–167 (2021). 10.1146/annurev-bioeng-090120-094330 33756087

[43] N. Matic , Prognostic value of hypoxia-responsive gene expression profile in patients diagnosed with head and neck squamous cell carcinoma. Transl. Oncol. **39**, 101841 (2024). 10.1016/j.tranon.2023.101841 PMC10687700 38016355

[44] A. I. Morrison, M. J. Sjoerds, L. A. Vonk, S. Gibbs, J. J. Koning, In vitro immunity: An overview of immunocompetent organ-on-chip models. Front. Immunol. **15**, 1373186 (2024). 10.3389/fimmu.2024.1373186 PMC11148285 38835750

[45] A. A. Bhat , Tumor microenvironment: An evil nexus promoting aggressive head and neck squamous cell carcinoma and avenue for targeted therapy. Signal Transduct. Target. Ther. **6**, 12 (2021). 10.1038/s41392-020-00419-w PMC7804459 33436555

[46] Z. Guo , The role of the tumor microenvironment in HNSCC resistance and targeted therapy. Front. Immunol. **16**, 1554835 (2025). 10.3389/fimmu.2025.1554835 PMC11996806 40236700

[47] Y. Gong , The tumor ecosystem in head and neck squamous cell carcinoma and advances in ecotherapy. Mol. Cancer **22**, 68 (2023). 10.1186/s12943-023-01769-z PMC10077663 37024932

[48] J. Huang , Extracellular matrix and its therapeutic potential for cancer treatment. Signal Transduct. Target. Ther. **6**, 153 (2021). 10.1038/s41392-021-00544-0 PMC8062524 33888679

[49] R. Millen , Patient-derived head and neck cancer organoids allow treatment stratification and serve as a tool for biomarker validation and identification. Med **4**, 290–310.e12 (2023). 10.1016/j.medj.2023.04.003 37178682

[50] R. Mooney, B. Tawil, M. Mahoney, Specific fibrinogen and thrombin concentrations promote neuronal rather than glial growth when primary neural cells are seeded within plasma-derived fibrin gels. Tissue Eng. Part A **16**, 1607–1619 (2010). 10.1089/ten.TEA.2009.0372 20028220

[51] L. Wullkopf , Cancer cells’ ability to mechanically adjust to extracellular matrix stiffness correlates with their invasive potential. Mol. Biol. Cell **29**, 2378–2385 (2018). 10.1091/mbc.E18-05-0319 PMC6233061 30091653

[52] E. Lopez-Vince, C. Wilhelm, T. Simon-Yarza, Vascularized tumor models for the evaluation of drug delivery systems: A paradigm shift. Drug Deliv. Transl. Res. **14**, 2216–2241 (2024). 10.1007/s13346-024-01580-3 PMC11208221 38619704

[53] S. Kim , 3D vascularized microphysiological system for investigation of tumor-endothelial crosstalk in anti-cancer drug resistance. Biofabrication **15**, 045016 (2023). 10.1088/1758-5090/acef99 37567223

[54] S. Faulkner, P. Jobling, B. March, C. C. Jiang, H. Hondermarck, Tumor neurobiology and the war of nerves in cancer. Cancer Discov. **9**, 702–710 (2019). 10.1158/2159-8290.CD-18-1398 30944117

[55] J. Burgess , Chemotherapy-induced peripheral neuropathy: Epidemiology, pathomechanisms and treatment. Oncol. Ther. **9**, 385–450 (2021). 10.1007/s40487-021-00168-y PMC8593126 34655433

[56] J. I. Kim , Human assembloid model of the ascending neural sensory pathway. Nature **642**, 143–153 (2025). 10.1038/s41586-025-08808-3 PMC12137141 40205039

[57] L. M. Terrier, N. Hadjikhani, C. Destrieux, The trigeminal pathways. J. Neurol. **269**, 3443–3460 (2022). 10.1007/s00415-022-11002-4 35249132

[58] J. P. Fredrikson , Engineering neuronal networks in granular microgels to innervate bioprinted cancer organoids on-a-chip. Lab Chip **25**, 3467–3481 (2025). 10.1039/d5lc00134j 40269972

[59] A. Cordiale , An Innovative “tooth-on-chip” microfluidic device emulating the structure and physiology of the dental pulp tissue. Adv. Healthcare Mater. **15**, e02080 (2025). 10.1002/adhm.202502080 PMC12790315 40838491

[60] S. Li, H. Xiao, Perineural invasion as a neuro−immune niche in head and neck cancer: Mechanisms of immune evasion and therapeutic implications. Front. Immunol. **16**, 1719675 (2025). 10.3389/fimmu.2025.1719675 PMC12722934 41445745

[61] L. B. Schmitd, C. S. Scanlon, N. J. D’Silva, Perineural invasion in head and neck cancer. J. Dent. Res. **97**, 742–750 (2018). 10.1177/0022034518756297 PMC6728584 29443582

[62] A. Jedlinski, A. Ansell, A.-C. Johansson, K. Roberg, EGFR status and EGFR ligand expression influence the treatment response of head and neck cancer cell lines. J. Oral Pathol. Med. **42**, 26–36 (2013). 10.1111/j.1600-0714.2012.01177.x 22643066

[63] S. C. D. Hartogh , Dual reporter MESP1mCherry/w-NKX2-5eGFP/w hESCs enable studying early human cardiac differentiation. Stem Cells **33**, 56–67 (2015). 10.1002/stem.1842 25187301

[64] F. R. Boroojeni , Proteolytic remodeling of 3D bioprinted tumor microenvironments. Biofabrication **16**, 025002 (2023). 10.1088/1758-5090/ad17d1 38128125

[65] J. Schindelin , Fiji: An open-source platform for biological-image analysis. Nat. Methods **9**, 676–682 (2012). 10.1038/nmeth.2019 PMC3855844 22743772

[66] A. M. Pignataro , An innervated and vascularized HNSCC-on-a-chip model built on defined and tunable engineered extracellular matrices. Gene Expression Omnibus. https://www.ncbi.nlm.nih.gov/geo/query/acc.cgi?&acc=GSE336833. Deposited 29 June 2026. 10.1073/pnas.2602541123 PMC13732190 42945260

