# Supplementary material for: An innervated and vascularized HNSCC-on-a-chip model built on defined and tunable engineered extracellular matrices
Source: Proc Natl Acad Sci U S A. 2026 Oct 27;123(44):e2602541123. doi: 10.1073/pnas.2602541123 (PMC13732190; doi:10.1073/pnas.2602541123)
Supplement: Supplementary file 1 [file pnas.2602541123.sapp.pdf]

Supplementary Figure S1

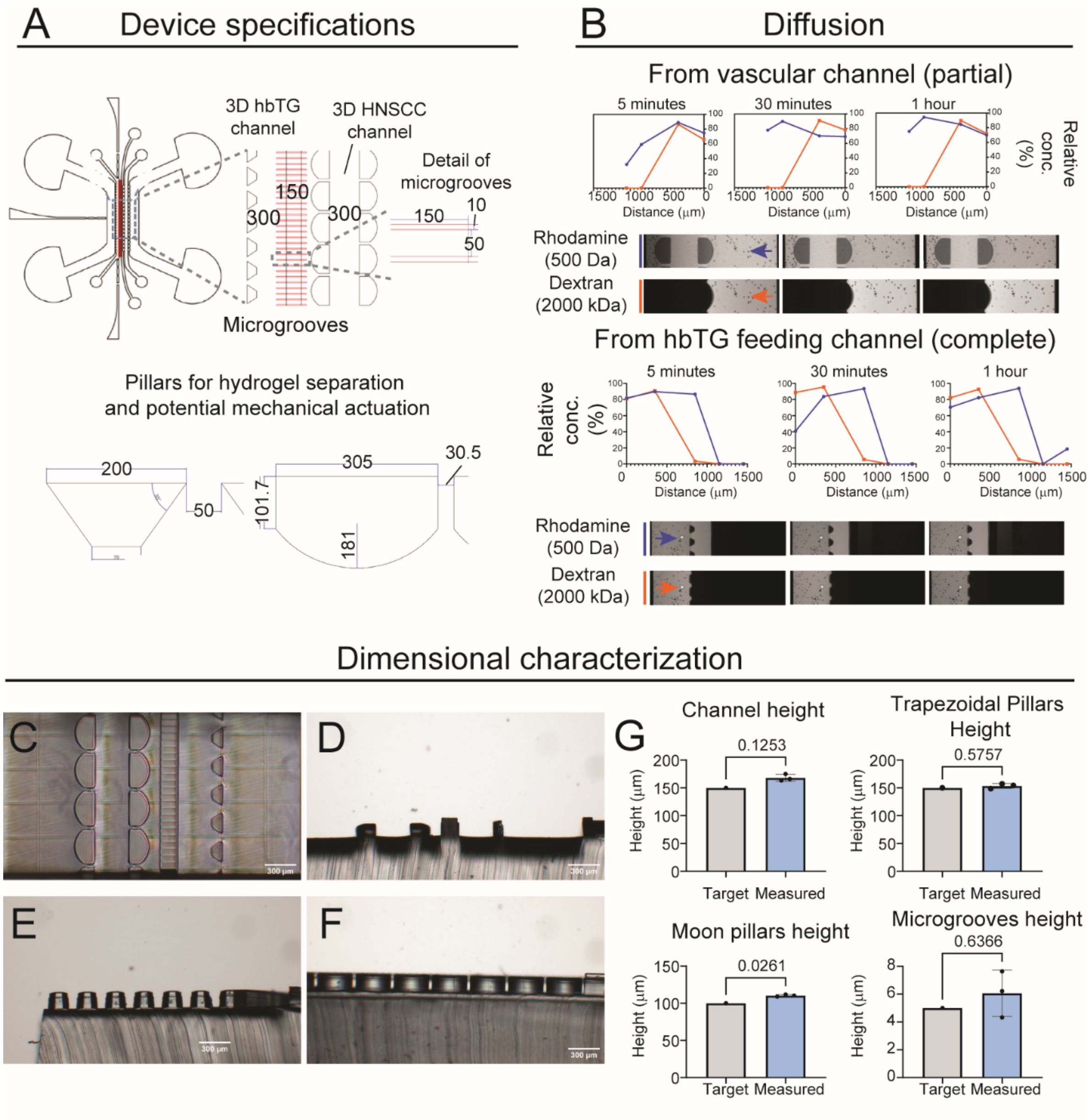

Supplementary Figure S1. A) Details of the device design. All dimensions are expressed in μm. **B)** Diffusion characterization of the microfluidic devices using fluorescent tracers (i.e. Rhodamine B (500 Da) and Dextran (2000 kDa)). Top panels show the diffusion of rhodamine B (blue line) and dextran (red line) from the vascular channel into the adjacent 3D HNSCC compartment in the partial device, while bottom panels show the diffusion from the trigeminal medium channel into the trigeminal ganglion and 3D HNSCC compartments in the complete device. Representative fluorescence microscopy images and corresponding percentage of the relative concentration profiles are shown for Rhodamine B and dextran at 5 minutes, 30 minutes, and 1 hour. Blue and red arrows indicate the diffusion direction of rhodamine and

dextran, respectively .Complete design top layer technical characterization. C). Pillars and microgrooves' top view. D) 3D culture channels cross-section. E) Trapezoidal pillars side view. F) moon-shaped pillars side view. G) Statistical analysis of the desired (Target) and obtained (Measured) chips dimensions. N = 3, t-test.

Supplementary Figure S2

HABCN Batch 4

% DS = 12,33%

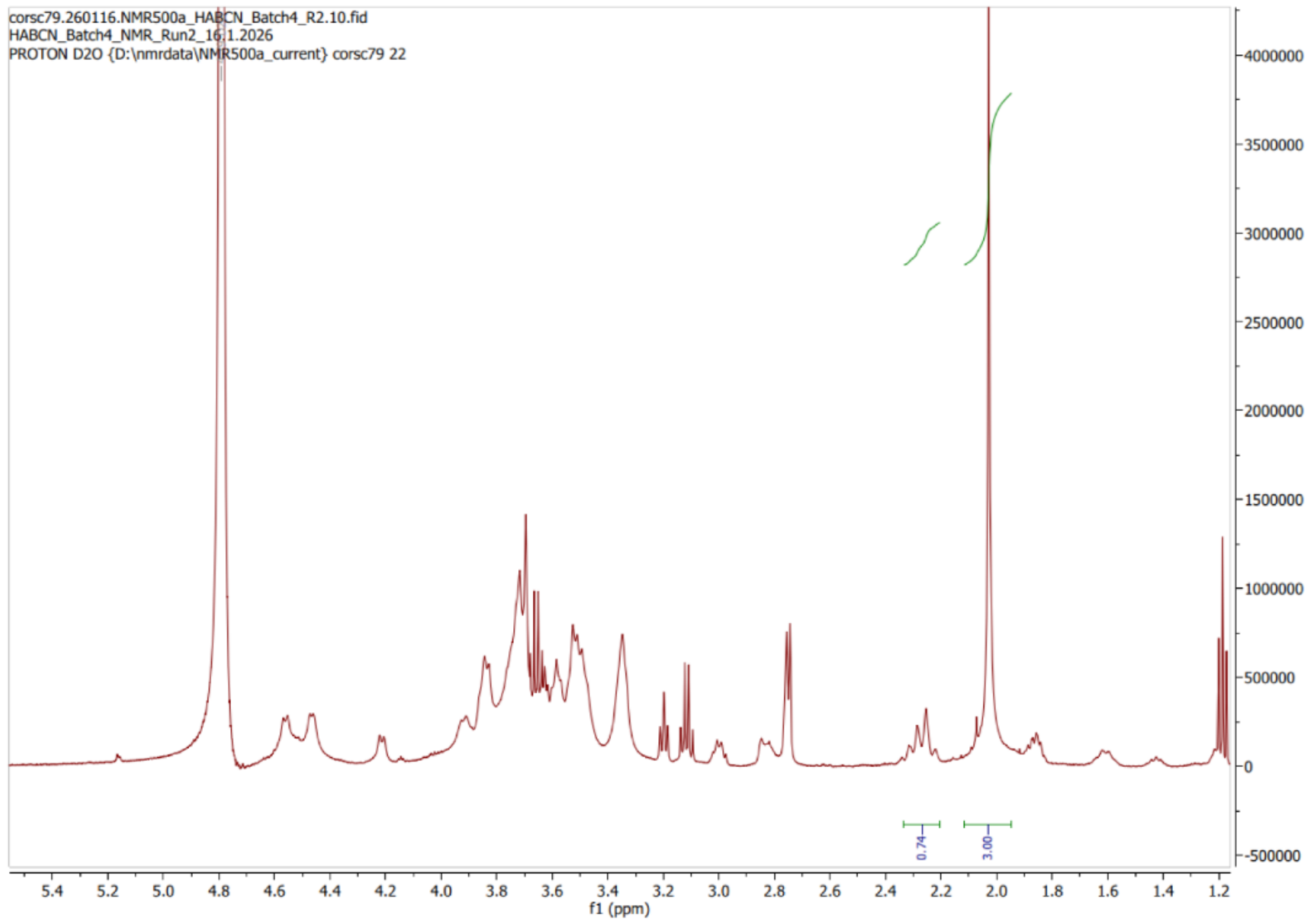

HABCN Batch5

% DS = 15,67%

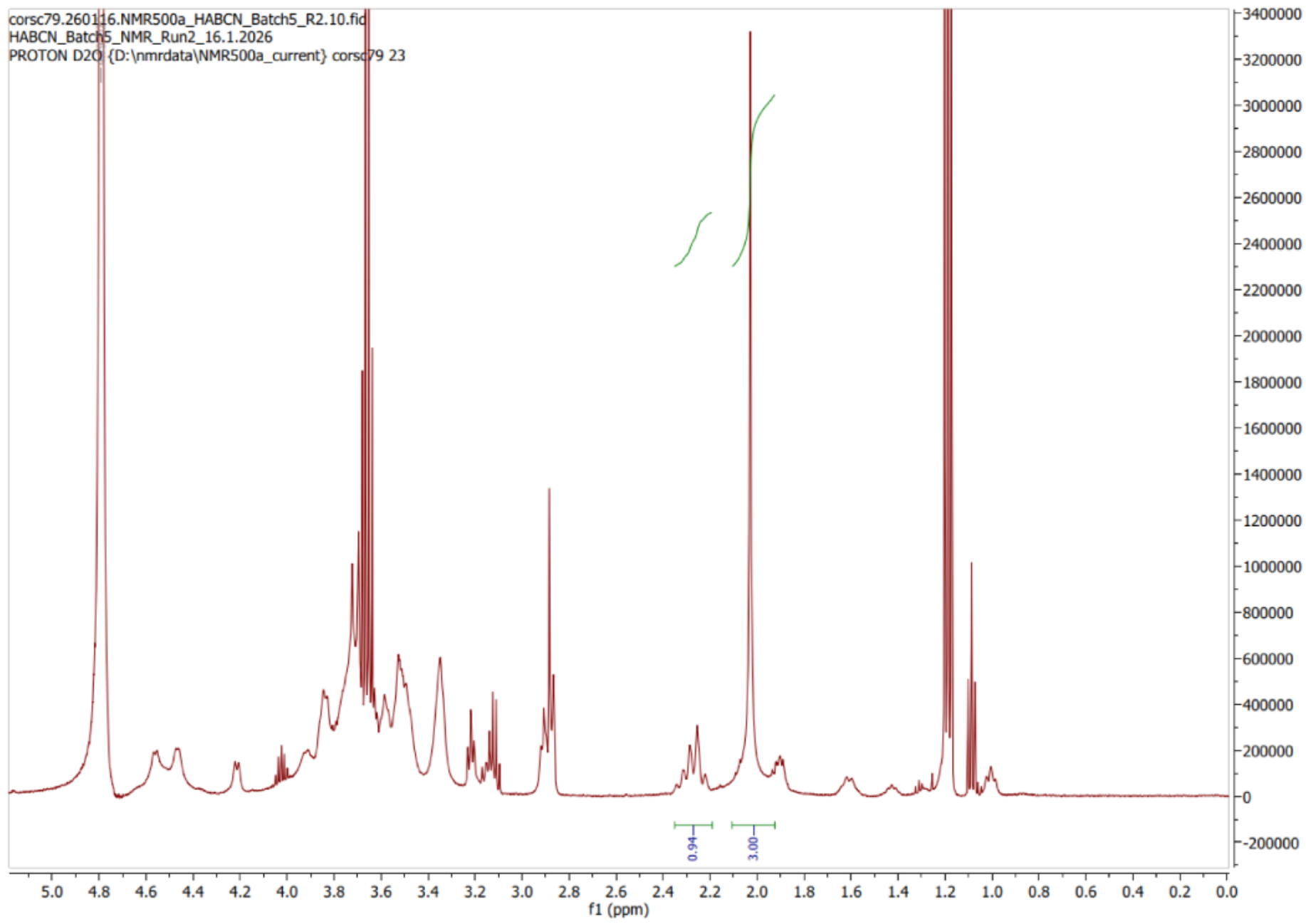

Supplementary Figure 2. ^{1}H-NMR spectra of HA-BCN batches.

Supplementary Figure S3

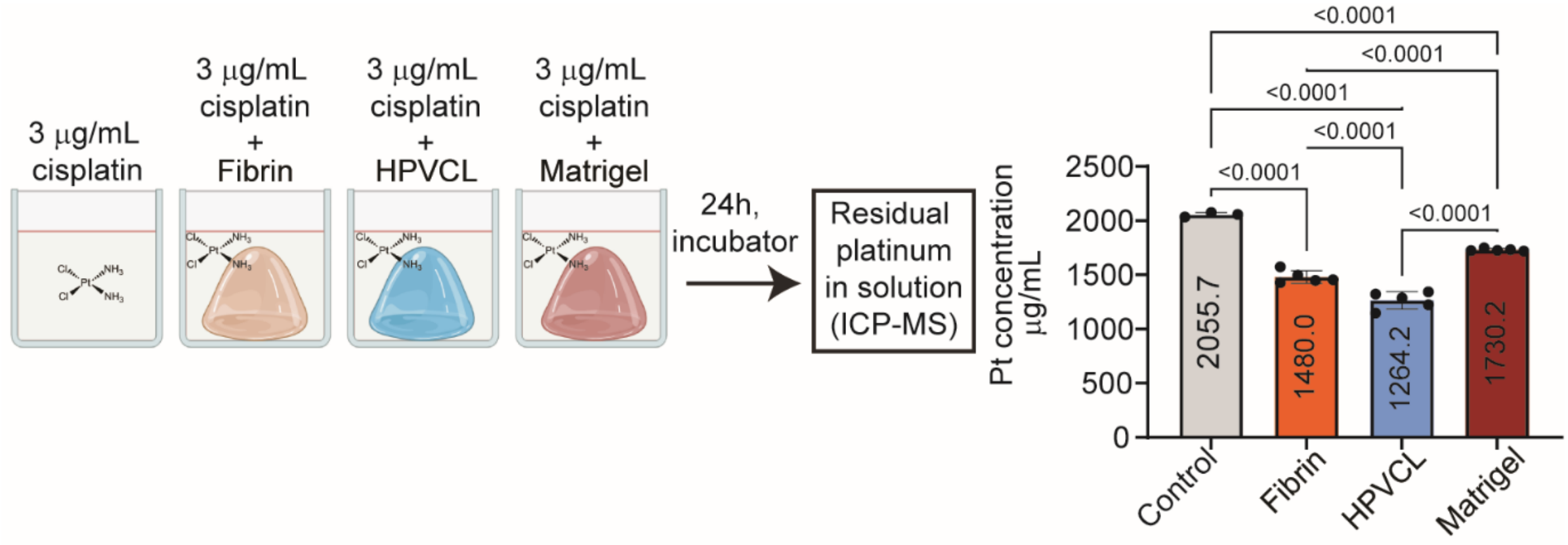

Supplementary Figure S3. Engineered extracellular matrices differentially retain cisplatin. Cell-free fibrin, HPVCL, or Matrigel hydrogels were incubated with 3 µg/mL cisplatin in PBS for 24 h, after which the platinum concentration remaining in the surrounding solution was quantified by inductively coupled plasma mass spectrometry (ICP-MS). Data are presented as mean ± SD (n = 5 for all conditions, except control, for which n = 3). Statistical significance was determined by one-way ANOVA with Tukey's multiple comparisons test.

Supplementary Video S1

See attached file

Supplementary Video S1. ATP-evoked calcium responses in human bioengineered trigeminal ganglia (hbTG). Live calcium imaging of day-40 hbTG expressing the genetically encoded calcium indicator jGCaMP8f following stimulation with 1 mM ATP. hbTG were transduced with AAV9-CAG-jGCaMP8f-WPRE on day 34 of differentiation in the engineered extracellular matrix.

Supplementary Materials and Methods

Microfluidic devices design and fabrication

The microfluidic device layout was designed using computer-aided design (CAD) software (AutoCAD®, AutoDesk Inc.). It consists of two main compartments for 3D cell-laden hydrogel cultures, one dedicated to the trigeminal compartment (hbTG compartment) and the other to the target tissue, represented by the 3D HNSCC model (HNSCC compartment). Both compartments are 150 μm high and 700 μm wide, and they are connected by 115 microgrooves ((Supplementary Figure S1). These microgrooves were specifically designed to allow axonal growth while preventing the migration of neuronal cell bodies. Each microgroove measures 150 μm in length, 5 μm in height, and 10 μm in width, with a 50 μm spacing between them. The hbTG compartment is a single-channel chamber with an inlet (⌀ = 1 mm) for 3D hbTG gel injection and an external channel with two reservoirs (⌀ = 5 mm) for medium supply. Similarly, the HNSCC compartment is a single-channel chamber, featuring gel injection ports (⌀ = 1 mm) to accommodate HNSCCs, CAFs, and HUVECs resuspended in the eECM of choice. The vascular compartment, responsible for supplying medium to the tumoral tissue, includes two reservoirs (⌀ = 5 mm) that serve as inlets for HUVECs seeding. The pillars in the trigeminal compartment have a trapezoidal cross-section, with a maximum length of 200 μm and a minimum inter-pillar distance of 50 μm. In contrast, the target tissue compartment features moon-shaped pillars, measuring 305.1 μm in length with 30.5 μm spacings between them. The pillars' geometry, size, and spacing were specifically designed to retain the hydrogel upon injection (1). In order to systematically optimize the two compartments, two models were designed: a complete device and a partial device. The complete design integrates both the target tissue compartment and the innervation compartment.

Devices fabrication

The devices are entirely made of PDMS (Sylgard 184; Dow Corning) and consist of two main components: a top microfluidic layer, where the channel structures are engraved, and a flat bottom layer. Their fabrication was carried out using photolithography and soft lithography techniques. In particular, the process began with the creation of a silicon master mold in a cleanroom environment (PoliFAB, Politecnico di Milano) using maskless photolithography. A 5 μm-thick negative photoresist layer (Cat. #SU-8 2005; MicroChem, USA) was spin-coated onto a 4'' silicon wafer, followed by the polymerization of microgrooves through direct laser writing (Heidelberg MLA100, Heidelberg Instruments). The non-cross-linked photoresist was

dissolved using a developing solution (SU-8 developer; MicroChem), the substrate was dried, and two layers of negative photoresist respectively 50 µm and 100 µm thick (Cat. #SU-8 2035, Cat. #SU-8 2100; MicroChem, USA) were sequentially spin-coated and cross-linked, resulting in a 150 µm-thick chamber layer. Following the exposure process, the non-cross-linked photoresist was eliminated using the developing solution, leaving behind a negative mold of the desired PDMS microfluidic layer. The features of the wafer were then transferred onto a PDMS stamp, which underwent technical characterization before being used to produce multiple copies of the master molds in epoxy resin (C-SYSTEMS 10 10 CFS). To create the new master molds, component A and component B were mixed in a 2:1 (v/v) ratio, stirred, and degassed using a vacuum pump. Once degassed, the resin was poured onto the PDMS master mold, which had been previously placed inside an aluminum Petri dish with its microstructures facing upwards. The Petri dish was transferred into the vacuum pump for additional degassing. Afterward, the resin was placed on a flat surface and left to cure overnight. The next day, the PDMS master mold was carefully removed using a spatula, and the resin underwent exposition to silane vapors for 20 minutes in order to enhance its surface properties. At this point the resin was used to produce multiple PDMS replicas, each containing three independent microfluidic units, allowing three technical replicates to be performed within the same chip. The final PDMS replicas of the devices were fabricated by mixing the pre-elastomer base with a curing agent in a 10:1 weight ratio. After degassing, the PDMS was poured onto the resin mold and degassed again. After curing at 65°C for 2.5 hours, to produce the chips, the PDMS top layers were peeled off from the resin molds and bonded via air plasma treatment (Harrick Plasma Inc.) onto either a glass coverslip (Ted Pella, 24 × 60 mm) or a flat PDMS layer. Before bonding, holes were created in the top layer using 5 mm biopsy punchers for reservoirs and 1 mm biopsy punchers for the inlets and outlets. The assembled device was then cured at 65°C for 20 minutes to ensure stable bonding. After bonding and before use, devices were visually inspected under a stereomicroscope to verify the integrity of channels, reservoirs, pillars, and microgrooves. Devices showing incomplete bonding, mispunched reservoirs, collapsed or deformed channels, incomplete replication of pillars or microgrooves, and persistent trapped air bubbles, were discarded. Only suitable devices were sealed and autoclaved before use.

Technical characterization

To ensure that the fabricated PDMS replicas met the intended dimensional specifications, key features such as the central channel height, pillars height, and width were analysed. First, the devices were divided into three sections, with each section containing a single microfluidic

unit. Each unit was then further sectioned transversely at the pillar and microgrooves' locations to obtain three thin slices. The samples were examined under a microscope, and images were captured using a digital camera. The dimensional characterization of the devices confirmed reproducible fabrication of the main microfluidic features, and no significant differences were observed between target and measured dimensions for the main channel height, trapezoidal pillars, or microgroove height (Supplementary Figure S1). Overall, the fabrication process reproduced the intended microfluidic architecture with minor geometry-dependent deviations. Functional injectability tests showed that the HA-BCN/PEG-Az8-laminin hydrogel could be loaded into the 3D compartments, crosslinked within 30 min, and remained confined by the pillar arrays. Measurements were performed using Fiji/ImageJ (2) and AmScope Camera software (AmScope, UK), with three independent measurements taken per chamber, followed by the calculation of the mean value and the standard deviation.

Injectability tests

Once fabricated, the four chip designs underwent an injection test with a functionalized HA-PEG based hydrogel to ensure that the gel could be injected smoothly without lateral leakage. First, 1.5% w/v HA-BCN was mixed with mouse non-functionalised laminin (0.1 mg/mL final concentration) and then with 1.5% w/v PEG-Az8. HA-BCN and PEG-Az8 were mixed at 4:1 ratio. Subsequently the gel was injected into the 3D HNSCC and 3D hbTG channels of the devices and incubated at 37°C and 5% CO_2. After gelation, a suspension of red polystyrene microbeads (10 μm diameter, Sigma-Aldrich) in phosphate-buffered saline (PBS, Thermo Fisher) was inserted into the medium channels to assess possible leakage across the pillar arrays. Brightfield (BF) and phase contrast (PH) images of the channels were captured to assess the presence of the gel-liquid interface and videos of beads movement in the medium channels were recorded. Finally, image and video analysis was conducted using Fiji/ImageJ software (2). Fluorescent polystyrene microbeads introduced into the adjacent medium channels remained restricted to the fluidic compartment, confirming hydrogel retention and stable gel-liquid interfaces.

Dextran and rhodamine diffusion tests (Metabolites and Drug Molecules Mass Transport)

On the same microfluidic devices, experimental tests were conducted with molecules of different molecular weights to evaluate molecular transport and barrier permeability. Rhodamine B (i.e., 500 Da) and Dextran (i.e., 2000 kDa) were introduced into the reservoirs of the lateral medium channels to monitor their diffusion toward the adjacent target

compartments. Specifically, tracer transport was evaluated from the vascular channel toward the 3D HNSCC compartment across the pillar interface, and from the hbTG medium channel toward the hbTG compartment and across the microgrooves connecting the hbTG and HNSCC compartments. During the 1-hour experiment, fluorescence microscopy images were acquired at regular intervals and subsequently analysed using Fiji/ImageJ (2).

On-chip culture quality control and exclusion criteria

On-chip cultures were inspected after hydrogel loading, medium addition, HUVEC seeding, hbTG seeding, and throughout the culture period. Chips were excluded from downstream analyses if hydrogel leakage into the adjacent medium channel occurred, if the gel failed to crosslink or became unstable upon medium addition, if the gel dried during culture, or if detachment of the bonded glass coverslip led to loss of compartmentalization and cell invasion outside the intended channels. Vascularized chips were excluded when a continuous vascular channel failed to form, which could result from insufficient channel coating, impaired HUVEC attachment, or obstruction at the reservoir/channel entrance. Innervated chips were excluded when hbTG aggregates obstructed the inlet during seeding, preventing proper loading of the ganglia into the hbTG compartment and subsequent innervation.

Hydrogels composition and fabrication

The formulations of all the hyaluronan-based eECMs used in this work are reported in Supplementary Table S1.

The following reagents were used to produce Hyaluronic acid (HA) / PEG-based hydrogels: sodium hyaluronate (100-150 kDa HA, Cat. #HA100K-5, LifeCore Biomedical, Minneapolis, USA); 8-arm PEG azide (PEG-Az8, 10 kDa, Cat. #PSB-881-1g, Creative PEGWorks); N-(3-dimethylaminopropyl)-N0 - ethylcarbodiimide (EDC), 1-hydroxybenzotriazole hydrate, N-[(1 R,8S,9s)-Bicyclo[6.1.0]non-4-yn-9-ylmethyloxycarbonyl]-1,8-diamino-3,6-dioxaoctane (BCN-NH2, Cat. #745073-100MG; Merck), 2-(N-morpholino) ethanesulfonic acid (MES) (100 mM, pH 7), human laminin 521 (Cat. #LN521-05, BioLamina), PureCol (3.2 mg/mL, Cat. #5005-100 ML, Advanced Biomatrix). Hydrogels were prepared by mixing HA-BCN and PEG-Az8 at a 4:1 molar ratio. The total polymer stock concentrations used were 1% (w/v%), 1.5%, or 2%, with the volumes adjusted accordingly to maintain the desired ratios. Hydrogels were supplemented with bioactive functionalization: cRGD-Az (1 mM), Human Recombinant Laminin 521 (9 μg/mL), Murine Laminin (Cat. #L2020, Sigma-Aldrich), Collagen I (16-17 μg/mL, Cat. #A1048301, ThermoFisher Scientific, or Cat. #5005-100 ML, Advanced

Biomatrix); cyclic RGD (Arg-Gly-Asp) (cRGD-Az); protease-degradable crosslinker VPM-Az2, synthesized as previously described (3). Partial protease-dependent degradability was ensured by adding the degradable crosslinker VPM-Az_2 (2 mg/mL). Cells or trigeminal placodes were resuspended in HA-BCN at the desired concentration; the additional hydrogel components were then added to the HA-BCN-cell suspension, with Az-bearing crosslinking agents added at the end. Loaded hydrogels were cast as domes in well plates or injected into the dedicated channels of the organ-on-a-chip devices. Samples were incubated at 37 °C to allow hydrogel crosslinking before culture medium addition, with crosslinking times defined according to the specific hydrogel formulation and experimental application, as detailed in the corresponding sections.

Fibrin-based hydrogels

Human fibrinogen (100 mg/mL in 0.9% NaCl; Cat. # F3879-250MG, Sigma-Aldrich) and thrombin (100 U/mL in 40 mM $CaCl_2$; Cat. #T6884-100UN, Sigma-Aldrich) stock solutions were diluted to final working concentrations of 40 mg/mL and 10 U/mL, respectively. With fibrinogen and thrombin on-ice, cells were resuspended in thrombin. The two components were mixed at a 1:1 volume ratio immediately before use to initiate fibrin polymerization. Loaded fibrin hydrogels were allowed to solidify for 10 minutes at 37°C in the incubator, before adding culture medium.

Hyaluronic acid functionalization with BCN

HA was functionalized with BCN (HA-BCN) as follows. HA was dissolved in MES buffer (100 mM, pH 7, 40 mL) on an orbital shaker for 2 hours. 100 mg BCN-NH2 were dissolved in a mixture of acetonitrile and milliQ water (MQ) (5:1 v/v, 6 mL), 1-Hydroxybenzotriazole hydrate (HOBt, 83 mg), and 1-ethyl-3-(3-dimethylaminopropyl) carbodiimide hydrochloride (236 mg, EDC). The acetonitrile solution was added in fractions to the hyaluronic acid solution and dialyzed against acetonitrile (10% v/v) for 24 hours, MQ water for 7 days, and adjusted to pH 6.5. The solution was diluted 1:1 with MQ water and freeze dried for 2 days. Two batches of HA-BCN were used for this work. To determine the degree of BCN substitution to HA backbone (%DS), HA-BCN was resuspended in deuterium oxide and analyzed by ^{1}H-NMR (%DS batch4 = 12.33%; %DS batch5 = 15.67%. See Supplementary Materials for ^{1}H-NMR spectra).

HNSCCs-CAFs-HUVECs dome cultures

HNSCCs and CAFs were washed with PBS and dissociated using 0.25% trypsin/EDTA for 5 minutes at 37 °C, while HUVECs were treated with TrypLE Express for 4 minutes at 37 °C. The enzymatic reactions were neutralized by adding culture medium (2× the digestion volume), and the cell suspension was then centrifuged (800g, 3 minutes) and cells counted using a Countess 3 Automated Cell Counter (Thermo Fisher). The three cell types (HCHs) were mixed at a 1:3:1 ratio, centrifuged, and resuspended in the hydrogel of choice. The exact hydrogel composition, precursor concentrations, final component concentrations and mixing ratios, used for each experimental application are reported in table 1. For all hydrogel conditions, plates were inverted immediately after casting and kept upside down for 10 min at 37 °C and 5% CO_2 to prevent cell sedimentation and promote homogeneous cell distribution within the hydrogel. For the HA-BCN / PEG-based hydrogel (see table 1), cells were resuspended in HA-BCN to achieve a final cell density of 15 × 10^6 cells/mL. HA-BCN and cells were then sequentially supplemented, in this order, with laminin, type I collagen, and VPM-Az_2, followed by PEG-Az_8. The resulting HPVCL–cell suspension was incubated at 37 °C and 5% CO_2 until complete gel crosslinking was achieved after 30 minutes. For the fibrin-based condition (stock and final concentration listed in table 1), HNSCCs, CAFs, and HUVECs (HCHs) were mixed at a 1:3:1 ratio, centrifuged, and resuspended in the thrombin solution at a cell density of 30 × 10^6 cells/mL. Keeping both solutions on ice, equal volumes (5 µL each) of the thrombin–cell suspension and fibrinogen solution were combined to generate the fibrin–cell mixture with a final cell density of 15 × 10^6 cells/mL. The cell-gel suspension was then incubated at 37 °C and 5% CO_2 until complete gelation occurred (10 minutes). For the Matrigel-based condition, growth factor-reduced Matrigel (Cat. # 354230, Corning) was thawed on ice according to the manufacturer's instructions. HCHs were mixed at a 1:3:1 ratio, centrifuged, and resuspended in Matrigel at a final cell density of 15 × 10^6 cells/mL and kept on ice. For dome formation, the Matrigel-cell suspension was carefully dispensed and incubated at 37 °C and 5% CO_2 for approximately 45 min to allow complete gelation. Culture medium was then gently added to each well. From day 0 onward, dome cultures were maintained in a 50:50 mixture of supplemented EGM-2 and DMEM/F-12 medium. For fibrin-based domes, aminocaproic acid (ACA, 2 mg/mL; Cat. No. A7824; Thermo Fisher Scientific) was added to the culture medium to prevent fibrin degradation.

Cisplatin treatment on 3D gel domes

For cisplatin-treated dome cultures, treatment was initiated on day 1 after cell seeding and maintained until day 3. The culture medium was replaced once daily with medium containing

3 μg/mL cisplatin, resulting in a total exposure time of 48 h. Untreated dome cultures were maintained under the same medium replacement schedule without cisplatin.

ICP-MS

Residual cisplatin concentration was measured via ICP-MS (inductively coupled plasma mass spectrometry). To this end, cisplatin was diluted to a concentration of 3 μg/mL in PBS, and 300 μL of the resulting solution were incubated for 24 hours with 10 μl-domes of Matrigel, HPVCL, or fibrin, in a 24 well plate, in a humidified incubator (37 °C, 5% CO2, 90% humidity). The solution was then removed, diluted 75x and acidified with 1% suprapure nitric acid. The samples were then analyzed via an Agilent 8900 ICP-MS-instrument at m/z=195 using He addition to reduce interferences, to determine platinum concentration. Note that the expected platinum concentration is equal to approximately 65% of the expected cisplatin concentration (1 mg cisplatin contains 0.650 mg platinum).

RNA sequencing

HCH cultures consisting of HNSCC cells, cancer-associated fibroblasts (CAFs), and endothelial cells were cultured in HPVCL, fibrin, or Matrigel matrices and harvested for bulk RNA sequencing (n = 3 independent biological replicates per condition). RNA was isolated from intact multicellular constructs without prior separation of individual cellular compartments, allowing characterization of ECM-dependent transcriptional states at the level of the integrated tumor microenvironment. Libraries were generated and sequenced by Plasmidsaurus (USA) using a 3′ UMI-based RNA sequencing workflow. FASTQ file quality was assessed using FastQC v0.12.1. Reads were quality-filtered using fastp v0.24.0 with poly-X tail trimming, 3′ quality-based tail trimming, a minimum Phred quality score of 15, and a minimum read length of 50 bp. Quality-filtered reads were aligned to the reference genome using STAR v2.7.11, followed by coordinate sorting with samtools v1.22.1. PCR and optical duplicates were removed using UMI-based deduplication with UMIcollapse v1.1.0. Alignment quality metrics, strand specificity, and read distribution across genomic features were assessed using RSeQC v5.0.4 and Qualimap v2.3, and summarized using MultiQC v1.32. Gene-level quantification was performed using featureCounts from the subread package v2.1.1 with strand-specific counting, fractional assignment of multimapping reads, exon and 3′ UTR features, and grouping by gene_id. Gene counts were annotated with gene biotype and metadata extracted from the reference GTF file. Downstream analyses were performed in R using edgeR. Lowly expressed genes were filtered using filterByExpr, and expression values were

normalized using the trimmed mean of M values (TMM) method. Principal component analysis and sample–sample correlation analyses were performed using normalized logCPM values. Pairwise differential expression analyses were conducted between HPVCL and fibrin, HPVCL and Matrigel, and fibrin and Matrigel cultures using the quasi-likelihood framework implemented in edgeR. P-values were adjusted for multiple testing using the Benjamini–Hochberg procedure. Genes with $FDR < 0.05$ and $|log2FC| > 1$ were considered significantly differentially expressed. Gene set enrichment analysis was performed using fgsea. Ranked gene lists were generated from edgeR differential expression statistics and interrogated using Hallmark, Reactome, Gene Ontology Biological Process, and C6 oncogenic signatures from the Molecular Signatures Database. In addition, custom HNSCC-relevant signatures representing ERBB signaling, WNT signaling, NOTCH signaling, YAP mechanotransduction, cancer stem cell-associated markers, epithelial/resistance-associated programs, and inflammatory vascular states were used for pathway activity analyses. Heatmaps were generated from row-scaled normalized expression values using representative genes associated with the indicated biological processes and signaling pathways.

hESCs-derived 3D trigeminal ganglia

Differentiation of hESCs into trigeminal ganglion (TG) neurons followed a previously established protocol (4) originally developed for generating human iPSC-derived trigeminal neurons in 2D culture. The published differentiation protocol was used to generate trigeminal placode progenitors in 2D culture up to day 14. From this stage onward, the protocol was adapted to a 3D culture format by gently detaching the progenitors with 0.5 mM EDTA, embedding them in the HA-PEG-cRGD-laminin (HPRL) hydrogel formulation (Supplementary Table S1), and maintaining them in complete Neurobasal medium to promote further maturation within the 3D eECM. In detail, upon thawing hESCs were seeded in complete E8 at a density of 2.5×10^5 cells/cm^2 on vitronectin-coated T25 flasks. Once desired confluency was reached (day 0), the medium was replaced with E6 (Cat. #A1516401, ThermoFisher Scientific) or TeSR-E6 (Cat. #05990, STEMCELL Technologies) supplemented with SB431542 10 μM (Cat. #1614/10, R&D Systems) and BMP4 5 ng/mL (Cat. # 314-BPE-010, R&D Systems) (E6-SB). From day 2 to day 4, E6-SB was further supplemented with CHIR99021 600 nM (Cat. #72054, STEMCELL Technologies) (E6-SBC). On day 5, medium was replaced with E6 supplemented with SB431542 10 μM. After 14 days of differentiation, the resulting trigeminal precursors (TPP) were detached using 0.5 mM EDTA and resuspended in HA-BCN. The HA-BCN / TPP suspension was then supplemented with cRGD-Az_2, laminin

521, and PEG-Az_8 (Supplementary Table S1). Upon gelation (45 minutes), trigeminal precursors were then cultured within the 3D eECM in Neurobasal (Cat. #21103049, ThermoFisher Scientific) supplemented with 2% B27 (Cat. #17504044, ThermoFisher Scientific), NGF 50 ng/mL (Cat. #450-01-100UG, ThermoFisher Scientific), BDNF 50 ng/mL (Cat. #AF-450-02-10UG, ThermoFisher Scientific; Cat. #78005, STEMCELL Technologies), GDNF 50 ng/mL (Cat. #450-10-10UG, ThermoFisher Scientific; Cat. #78058, STEMCELL Technologies), DAPT 10 µM (Cat. #J65864.MA, ThermoFisher Scientific), L-glutamine 1mM (Cat. # A2916801, ThermoFisher Scientific), hereafter described as complete Neurobasal. Cultures were kept up to 40 days to generate human bioengineered trigeminal ganglia (hbTG). For calcium imaging, hbTG were infected with AAV9-CAG-jGCaMP8f-WPRE (2.6×10^{11} GC/hbTG; stock titer: 2.6×10^{13} GC/mL, Addgene) on day 34 of differentiation in eECM. On day 40, hbTG were treated with ATP 1 mM, and calcium influx was live imaged with a Zeiss CLSM. Videos were analyzed with Fiji/ImageJ (2).

Tumor size analysis

3D image stacks acquired using a Leica DMi8 widefield microscope were deconvolved using Huygens and converted to maximum-intensity projections in Fiji/ImageJ. Projected tumor area was quantified for each tumor-on-a-chip device using Fiji/ImageJ, with each chip considered an independent biological replicate. Tumor tissue was segmented from the combined GFP and actin channels after subtraction of the CD31 channel to exclude endothelial structures. A fixed intensity threshold was applied to all images, followed by manual inspection to remove obvious segmentation artifacts without altering true tumor boundaries. Tumor size distributions were analyzed using empirical cumulative distribution functions (ECDFs). ECDFs were computed separately for each chip and then averaged across the chips per condition. Distributional differences between conditions were quantified using pairwise Kolmogorov–Smirnov (KS) distances computed between all chip pairs. KS distances (D) are reported as effect sizes summarizing the maximum separation between ECDFs. For the experiments reported in Figure 3, tumor area was quantified separately within the original HNSCC compartment and the vascular compartment. Total tumor area was calculated as the sum of the areas detected in both compartments. The fraction invaded was defined as the tumor area detected within the vascular compartment divided by the total tumor area. To evaluate the effects of extracellular matrix composition and cisplatin treatment, compartmental tumor area metrics were analyzed using linear models with hydrogel (fibrin or HPVCL), treatment (untreated or cisplatin), their interaction, and patient as fixed effects. Tumor area within the HNSCC compartment, vascular

compartment area, and total tumor area were log10-transformed prior to analysis to account for right-skewed distributions, whereas fraction invaded was analyzed on the original scale. Statistical significance of model terms was assessed by analysis of variance (ANOVA) on the fitted models. For the experiments reported in Figure 5, tumor area was quantified separately within the original tumor compartment and the vascular compartment. Total tumor area was calculated as the sum of the areas measured in both compartments, and the fraction invaded was defined as the area detected within the vascular compartment divided by the total tumor area. To account for experimental batch effects, compartmental tumor area metrics were analyzed using linear models with condition and batch included as fixed effects. Statistical significance of model terms was assessed by analysis of variance (ANOVA) on the fitted models. Linear models were used to estimate the independent effects of hydrogel composition, treatment, and patient (Figure 3), or condition and experimental batch (Figure 5), while accounting for potential confounding variables within a unified statistical framework. Statistical analyses were performed in R (version 4.3.2).

Supplementary Tables

Table S1

Component	Stock conc.	Final conc.	V[μL] for (50 μL)
**HA-BCN / PEG-Az8 / VPM-Az2/ COL / LAM (HPVCL)**			
HA-BCN	20 mg/mL	14.24 mg/mL	35.6
PEG-Az8	20 mg/mL	3.56 mg/mL	8.9
Laminin521	0.1 mg/mL	0.5 μg/mL	0.25
Collagen I	3.2 mg/mL	16 μg/mL	0.25
VPM-Az2	20 mg/mL	2 mg/mL	5
**HA-BCN / PEG-Az8 / cRGD-Az/ LAM (HPRL - for hbTG)**			
HA-BCN	10 mg/mL	7.2 mg/mL	36.05
PEG-Az8	10 mg/mL	1.8 mg/mL	9
cRGD-Az	0.701 mg/mL	6.3 μg/mL	0.45
Laminin521	0.1 mg/mL	9 μg/mL	4.5
**HA-BCN / PEG-Az8 / COL / LAM (HPCL)**			
HA-BCN	20 mg/mL	15.82 mg/mL	39.55
PEG-Az8	20 mg/mL	3.96 mg/mL	9.9
Collagen I	3.2 mg/mL	17.6 μg/mL	0.275
Laminin521	0.1 mg/mL	0.55 μg/mL	0.275
**HA-BCN / PEG-Az8 / LAM (for injection tests)**			
HA-BCN	15 mg/mL	11.2 mg/mL	37.33 μL
PEG-Az8	15 mg/mL	2.8 mg/mL	9.33 μL
Laminin521	1.5 mg/mL	0.1 mg/mL	3.33 μL
**Fibrinogen / Thrombin (Fibrin)**			
Fibrinogen	40 mg/mL	20 mg/mL	25
Thrombin	10 U / mL	5 U / mL	25

Table S1: Hydrogels composition.

Table S2

Forward primers (5'-3')		Reverse primers (5'-3')	
hACTB_Fw	CATGTACGTTGCTATCCAGGC	*hACTB_Rv*	CTCCTTAATGTCACGCACGAT
hBDNF_Fw	GGCTTGACATCATTGGCTGAC	*hBDNF_Rv*	CATTGGGCCGAACTTTCTGGT
hBRN3A_Fw	GGGCAAGAGCCATCCTTTCAA	*hBRN3A_Rv*	CTGTTCATCGTGTGGTACGTG
hCALCA_Fw	CCTCCCGCCAGAGAGGT	*hCALCA_Rv*	GCACATAGTCCTGCACCAGT
hCD274_Fw	TGGCATTTGCTGAACGCATTT	*hCD274_Rv*	GGGAGAGCTGGTCCTTCAAC
hFN1_Fw	CGGTGGCTGTCAGTCAAAG	*hFN1_Rv*	AAACCTCGGCTTCCTCCATAA
hGAPDH_Fw	AGGGCTGCTTTTAACTCTGGT	*hGAPDH_Rv*	CCCCACTTGATTTTGGAGGGA
hHES1_Fw	TCAACACGACACCGGATAAAC	*hHES1_Rv*	GCCGCGAGCTATCTTTCTTCA
hHYAL1_Fw	CTTCAGCCCCAAGGTTGTCC	*hHYAL1_Rv*	CACTGACATCCACGTCCACA
hMMP2_Fw	AGGGAGCGCTACGATGGAG	*hMMP2_Rv*	AGGTGTTCAGGTATTGCACTG
hMMP9_Fw	TGTACCGCTATGGTTACACTCG	*hMMP9_Rv*	GGCAGGGACAGTTGCTTCT
hNGF_Fw	GGCAGACCCGCAACATTACT	*hNGF_Rv*	CACCACCGACCTCGAAGTC
hPAX3_Fw	AAAGGTGAAGAGGAGGAGGC	*hPAX3_Rv*	CTCAAAAGCACGCTCCAGTT
hPIEZO2_Fw	GTTTAGCATCAAGCGAGCCC	*hPIEZO2_Rv*	CATGGCTCCCACATCGTACC
hSCN10A_Fw	GCAAGATTGAGCAGCAAAGAGTA	*hSCN10A_Rv*	TCTTTGTTCCCTGCTTGGCA
hSIX1_Fw	CTGCCGTCGTTTGGCTTTAC	*hSIX1_Rv*	GCTCTCGTTCTTGTGCAGGT
hTGFB1_Fw	GGCCAGATCCTGTCCAAGC	*hTGFB1_Rv*	GTGGGTTTCCACCATTAGCAC
hTNF_Fw	CCTCTCTCTAATCAGCCCTCTG	*hTNF_Rv*	GAGGACCTGGGAGTAGATGAG
hTRPM8_Fw	CAGGCTCAGCATGAGGAACA	*hTRPM8_Rv*	CTTGCACACATTCTCCGTGG
hTRPV1_Fw	AGAGTCACGCTGGCAACC	*hTRPV1_Rv*	AGAGACTCTCCATCACACTGTC
hVEGFA_Fw	TAAGTCCTGGAGCGTTCCCT	*hVEGFA_Rv*	ACGCGAGTCTGTGTTTTTGC

Table S2. Primer sequences.

Table S3

Target	Cells	Host	Manufacturer	Catalogue #	Dilution
CD31	HUVECs	Mouse	Thermo Scientific	PIMA513188	1:10-20
Ki67	Proliferating cells	Rabbit	Thermo Scientific	MA5-14520	1:200
Cleaved Caspase-3	Apoptotic cells	Rabbit	Thermo Scientific	25128-1-AP	1:200
NEFL	Trigeminal neurons	Rabbit	Thermo Scientific	MA5-14981	1:100
NEFL	Trigeminal neurons	Mouse	Thermo Scientific	10242123	1:100
SOX10	Glial cells	Rabbit	Cell Signaling	89356	1:1000
CRGP	Peptidergic nociceptors	Rabbit	Bachem	T-4031	1:200
IB4 (biotinylated)	Non-peptidergic nociceptors	-	Life Technologies	I21214	1:200
NF-200	Large myelinated neurons	Mouse	Merck	N0142	1:500

Target	Fluorophore	Manufacturer	Catalogue #	Dilution
Rabbit	Alexa fluor 700	Thermo Fisher	A-21038	1:200
Mouse	Alexa fluor 647	Thermo Fisher	A-31571	1:200
Rabbit	Alexa fluor 568	Thermo Fisher	A-10042	1:200
Biotin	Alexa fluor 488, streptavidin-conjugated	Thermo Fisher	S11223	1:500

Table S3. List of antibodies used for immunofluorescent staining.

References

1. P. Occhetta, *et al.*, Hyperphysiological compression of articular cartilage induces an osteoarthritic phenotype in a cartilage-on-a-chip model. *Nat. Biomed. Eng.* **3**, 545–557 (2019).

2. J. Schindelin, *et al.*, Fiji: An open-source platform for biological-image analysis. *Nat. Methods* **9**, 676–682 (2012).

3. F. R. Boroojeni, *et al.*, Proteolytic remodeling of 3D bioprinted tumor microenvironments. *Biofabrication* (2023). https://doi.org/10.1088/1758-5090/ad17d1.

4. B. Zimmer, *et al.*, Human iPSC-derived trigeminal neurons lack constitutive TLR3-dependent immunity that protects cortical neurons from HSV-1 infection. *Proc. Natl. Acad. Sci. U. S. A.* **115**, E8775–E8782 (2018).
